# Learning and inference with correlated neural variability

**DOI:** 10.1093/pnasnexus/pgaf284

**Published:** 2025-10-10

**Authors:** Yang Qi, Zhichao Zhu, Yiming Wei, Lu Cao, Zhigang Wang, Jie Zhang, Wenlian Lu, Jianfeng Feng

**Affiliations:** Institute of Science and Technology for Brain-Inspired Intelligence, Fudan University, Shanghai 200433, China; Key Laboratory of Computational Neuroscience and Brain-Inspired Intelligence (Fudan University), Ministry of Education, China; MOE Frontiers Center for Brain Science, Fudan University, Shanghai 200433, China; Institute of Science and Technology for Brain-Inspired Intelligence, Fudan University, Shanghai 200433, China; Key Laboratory of Computational Neuroscience and Brain-Inspired Intelligence (Fudan University), Ministry of Education, China; Institute of Science and Technology for Brain-Inspired Intelligence, Fudan University, Shanghai 200433, China; Centre for Integrative Neuroimaging, FMRIB, Nuffield Department of Clinical Neurosciences, University of Oxford, Oxford OX3 9DU, United Kingdom; Intel Labs China, Beijing 100190, China; Intel Labs China, Beijing 100190, China; Institute of Science and Technology for Brain-Inspired Intelligence, Fudan University, Shanghai 200433, China; Key Laboratory of Computational Neuroscience and Brain-Inspired Intelligence (Fudan University), Ministry of Education, China; Institute of Science and Technology for Brain-Inspired Intelligence, Fudan University, Shanghai 200433, China; Key Laboratory of Computational Neuroscience and Brain-Inspired Intelligence (Fudan University), Ministry of Education, China; Ji Hua Laboratory, Foshan 528200, China; Institute of Science and Technology for Brain-Inspired Intelligence, Fudan University, Shanghai 200433, China; Key Laboratory of Computational Neuroscience and Brain-Inspired Intelligence (Fudan University), Ministry of Education, China

**Keywords:** spiking neural network, moment closure, neural correlation, gradient-based learning

## Abstract

The abundance of both input and process noises in the brain suggests that stochasticity is an integral part of neural computing, but how spiking neural networks (SNN) can learn general tasks under correlated variability remain unclear. In this work, we propose a stochastic neural computing (SNC) theory to implement gradient-based learning in SNN in the noise-driven regime using a moment closure approach. This leads to a new class of deep learning architecture called the moment neural network (MNN), which naturally generalizes rate-based neural networks to second-order statistical moments. Once trained, the parameters of the MNN can be directly used to recover the corresponding SNN without further fine-tuning. The trained model captures realistic firing statistics of biological neurons, including broadly distributed firing rates and Fano factors as well as weak pairwise correlation. The joint manipulation of mean firing rate and correlation structure leads to a distributed neural code that maximizes task accuracy while simultaneously minimizing prediction uncertainty, resulting in enhanced inference speed. We further demonstrate the application of our method on Intel’s Loihi neuromorphic hardware. The proposed SNC framework offers insight into how SNNs process uncertainty and a practical way to build biologically plausible neural circuit models with correlated variability.

Significance Statement Despite the prevalence of noise in the brain, existing approaches to training spiking neural networks (SNN) such as artificial neural network (ANN)-to-SNN conversion and backpropagation-through-time are primarily designed for noise-free settings. Built on the principle of stochastic neural computing, the moment closure approach in this work enables gradient-based learning in SNN when membrane potential dynamics are driven strongly by noise and simultaneously provides an analytical tool capturing the propagation of correlated neural variability. The proposed method could be particularly useful for building neural circuit models to study the functional role of correlated variability in the brain and guide future development of brain-inspired intelligence and neuromorphic engineering.

## Introduction

Stochasticity is a hallmark of neural computation in the brain across input, neuronal, and behavioral levels (1)—from odorant molecules dispersed through turbulent airflow to complex tactile patterns, from highly irregular spiking activity of cortical neurons to pairwise correlations (2–5), and from trial-to-trial variability in behavioral responses to subjective uncertainty (6–8). The abundance of both input and process noises in the brain has led to the prominent idea that noise is an integral part of the computational process in the brain rather than an undesirable side effect (1, 9, 10). Stochastic neural dynamics is implicated in a broad range of brain functions from sensory processing (11, 12), sensorimotor control (13, 14), to uncertainty representation (15, 16), probabilistic inference (17–23), and neural population coding (24–27). The ability to represent and compute with uncertainty is thus a key aspect of biological intelligence which separates it from the deterministic, digital computing architectures of today (28).

An important feature of fluctuating neural activity in the brain is its rich noise correlation structures, with profound implications in both neural dynamics and functions (8, 24, 25, 27, 29). For dynamics, noise correlation can become coupled to mean firing rate through nonlinear spike emission (5). For functions, correlation structures can significantly impact the quality of neural codes (30–32), the saturation of information with population size (31, 33) and decision making processes (21, 34). Despite that pairwise neural correlations in the cortex are typically weak, they can lead to strong effects in the collective state of neural population (30).

Despite its importance in neural systems, the effect of stochasticity and correlated variability is often neglected in studies of learning in spiking neural networks (SNNs) and there lacks a method for training fluctuation-driven SNNs with generic synaptic weights performing arbitrary tasks. Existing gradient-based approaches to training SNNs can largely fall into two categories: ANN-to-SNN conversion and direct SNN training methods (35, 36). In ANN-to-SNN conversion, a continuous-valued artificial neural network (ANN) is pretrained using standard backpropagation algorithms and then converted to an SNN model by mapping the ANN parameters onto SNN. The conversion can be achieved through various post-training optimization techniques (37–43). The mapping from ANN to SNN are typically designed in an ad hoc manner for the sole purpose of model performance and there is a lack of theoretical link between SNN and continuous-valued ANN. Importantly, it remains unclear how a pretrained ANN model can account for various noise sources present in SNNs. Direct approaches to training SNN, on the other hand, use backpropagation-through-time (BPTT) to optimize the parameters of an SNN directly (36, 44, 45). This requires formulating the SNN as an equivalent recurrent neural network with binary spike inputs. The main challenge of direct training is due to the discontinuous, nondifferentiable nature of spike generation. Diverse methods such as surrogate gradient and spike time coding have been proposed to overcome this problem (36, 40, 44–54). However, BPTT-based techniques are designed primarily for training SNN in the noise-free regime and are unsuitable for training SNNs when neural activity is strongly driven by noise.

Given the important roles of stochasticity and correlated variability in neural processing, it is necessary to develop an alternative approach to training SNNs in the fluctuation-driven regime accounting for correlated variability. The main challenges lie in the nonlinearity and the high dimensionality of joint probability distribution of neural spiking activity. To overcome these challenges, we turn to a dimension reduction approach known as moment closure which leads to a closed and self-consistent set of ordinary differential equations involving only the statistical moments of a system (55). In neural systems, moment closure has been successfully derived under a range of settings through master equation (56, 57), system size expansion (58, 59), fluctuation expansion (57, 60), path integral (61, 62) and Fokker–Planck formalisms (63–70), though many of them only constitute a partial moment closure, e.g. assuming uncorrelated or Poisson firing statistics. In this work, we follow the moment neural network (MNN) developed in Refs. (65, 66) featuring a full moment closure of SNN with correlated variability. In essence, the MNN provides a minimalistic yet rich description of the statistical properties of an SNN up to second-order moments. For spiking neuron models, moment closure has been widely used to analyze the firing properties of neural population (64), balanced states in excitation–inhibition networks (71–73) and correlated neural variability (5, 31, 71) but have not seen wider adoption in the brain-inspired intelligence literature. This is largely due to the mathematical and computational complexity of the MNN which makes them difficult to scale up for learning general tasks. To overcome this, we employ an efficient numerical implementation of the MNN recently developed by us (74), allowing for a rapid evaluation of the moment mappings as well as their gradients. Importantly, this method enables the full expression of firing variability, as opposed to commonly assumed Poisson firing.

To develop our approach to learning in SNN with correlated variability, we first introduce an overall theoretical framework of stochastic neural computing (SNC) to illustrate how high-dimensional joint probability distributions of neural activity can be propagated and transformed through layers of nonlinearly coupled spiking neural populations. Next, we consider a concrete example of a feedforward SNN and apply moment closure to arrive at the corresponding MNN which captures the propagation of correlated variability across layers. We then implement gradient-based learning in MNNs by systematically generalizing components in conventional deep learning to second-order statistical moments. This yields a new class of deep learning architecture that, unlike conventional rate-based ANNs, explicitly incorporates and optimizes nonlinearly coupled, correlated neural variability. The synaptic weights obtained this way can be used directly, without further fine tuning of free parameters, to recover the original spiking neural network.

To demonstrate, we train an SNN to perform an image classification task using a feedforward architecture. Through minimizing a generalized cross entropy, the model is able to learn the task while simultaneously minimizing trial-to-trial variability of model predictions. The trained network naturally exhibits realistic properties of cortical neurons including mean-dominant and fluctuation-dominant activities as well as weak pairwise correlations. We reveal concurrent and distributed processing of signal and noise in the network and explain how structured neural fluctuations lead to both accurate inference and enhanced speed. We further demonstrate applications of the proposed method on neuromorphic hardware and explain how SNC may serve as a guiding principle for future design of neuromorphic computing.

## Results

### A probabilistic interpretation of spike-based neural computation

**Fig. 1.**
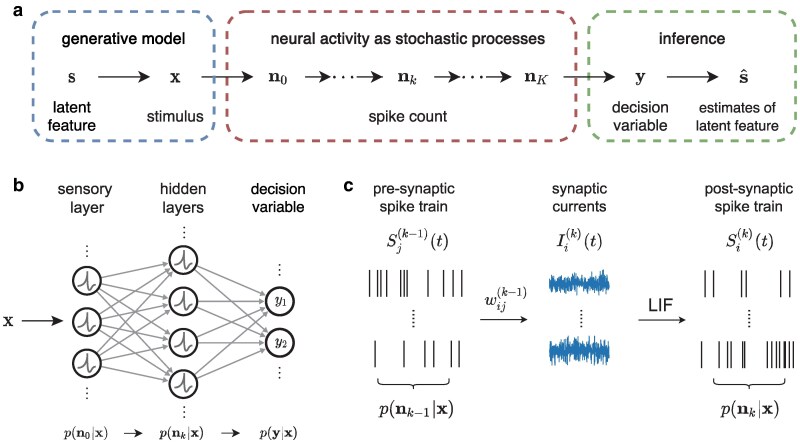
Spike-based stochastic neural computing (SNC). a) A graphical model for SNC which consists of a generative model describing the external environment, multiple stages of fluctuating neural activity states, and a readout for making inference. The index *k* represents different neural populations in a feedforward network or alternatively time in a recurrent network. b) Schematics of a spiking neural network implementing the computational processes outlined in (a), with each layer characterized by a joint probability distribution of neural spike count. c) Propagation of irregular neural spike trains through two feedforwardly connected neural populations. The presynaptic spike trains first undergo synaptic summation to generate fluctuating synaptic currents, which in turn drive postsynaptic neurons to fire. The probability distribution of spike count is transformed in a nontrivial way due to the nonlinear coupling of correlated neural variability.

We first establish a conceptual framework through a probabilistic interpretation of the computational processes in a generic feedforward neural network. Consider a computational process shown in Fig. 1a consisting of three components. The first component is a generative model that describes how an observable stimulus x in the environment depends on its latent features s. The second component is a model describing the fluctuating activity states $n_{k}$ of a group of neural populations, which are interpreted as random spike counts over a time window Δt. The index *k* represents different neural populations in a feedforward network or alternatively discrete time steps in a recurrent network. The last component is a decision variable or readout y used for producing estimates $\hat{s}$ about the latent features s.

To express these computation stages concretely, we write down the distribution $p(n_{k}|x)$ of the neural population state $n_{k}$ at each stage *k* in terms of the marginalization of its conditional probability over $n_{k-1}$ in the preceding stage as


(1) $$p(n_{k}|x)=\int p(n_{k}|n_{k-1})p(n_{k-1}|x)dn_{k-1}.$$

Note that the spike count time window is assumed to be sufficiently large relative to the temporal correlation timescale so that $p(n_{k}|n_{k-1})$ does not involve spike count at earlier times. By chaining Eq. 1 iteratively, we recover the probability of the readout y conditioned on the stimulus x


(2) $$p(y|x)=\int p(y|n_{K})\prod_{k=1}^{K}p(n_{k}|n_{k-1})p(n_{0}|x)dn_{0}\cdots dn_{K}.$$

Equipped with this conceptual framework, we can now define SNC as a series of neural operations [Eq. 1] that generates a desired conditional distribution p(y|x) of the readout y given a stimulus x. Under this view, the fundamental computing unit of SNC is the probability distribution of the activity state of a neural population p(n), and the basic operation of SNC is the transformation of these distributions across populations of neurons. In general, the transition probability $p(n_{k}|n_{k-1})$ depends on the model parameters (synaptic weights), which can be optimized through learning such that the model can generate correct estimates $\hat{s}$. In previous studies of probabilistic neural computation, the transition probability $p(n_{k}|n_{k-1})$ is often explicitly defined as independent Poisson or binary distributions with a firing probability depending on the input (75, 76). However, by making this simplification, these models essentially erase the correlation structures that naturally arise from synaptic coupling. For spiking neural networks with realistic firing dynamics, the high-dimensional joint transition probability $p(n_{k}|n_{k-1})$ generally does not have simple expressions.

A spiking neural network implementing this computational process is illustrated in Fig. 1b where each neuron in the network is modeled as a leaky integrate-and-fire (LIF) neuron (see Eqs. 6 and 7 in Methods section). One step of SNC carried out across two populations of spiking neurons is shown in more detail in Fig. 1c. As irregular spike trains from the presynaptic population converge at postsynaptic neurons, they give rise to fluctuating synaptic currents and subsequently irregular spike emissions in the postsynaptic neurons (5). Importantly, since these fluctuating synaptic currents are generated from a common pool of presynaptic neurons, they inevitably become correlated even if the input spikes are not. These correlated neural fluctuations are further transformed in a nonlinear fashion as they propagate across downstream neural populations.

To perform any useful computation, the spiking neural network needs to learn the set of parameter values *θ* that matches the readout distribution p(y|x;θ) with a desired target distribution $p^{*}(y|x)$. The probabilistic interpretation of the readout y allows us to design learning objectives (loss functions) in a principled manner (76). Here, we prescribe two such loss functions for regression and classification tasks under the supervised learning setting. For regression problems, a natural choice is the negative log likelihood


(3) $$L(\theta )=-\sum_{x\in D}\log\;p(y^{*}|x;\theta ),$$

where $p(y^{*}|x;\theta )$ is the likelihood of the network parameters *θ* for when $y=y^{*}$ with $y^{*}$ representing target output. For classification problems, class prediction is obtained by taking the class label *i* corresponding to the largest entry of y. The probability that the model predicts class *i* for a given input x can be expressed as $q_{i}(\theta )=\int p(y|x;\theta )1_{D_{i}}(y)dy$, where the indicator function $1_{D}(y)$ is equal to one if y∈D and zero otherwise, and $D_{i}$ denotes the set of all y whose largest entry is $y_{i}$. Denoting *t* as the target class, the goal is then to maximize the probability of correct prediction $q_{t}$. This leads to the loss function


(4) $$L(\theta )=-\sum_{x\in D}\log\;(q_{t})=-\sum_{x\in D}\int p(y|x;\theta )1_{D_{t}}(y)dy.$$

Interestingly, the same expression can be alternatively obtained from cross entropy $H(\theta )=-\sum_{i}p_{i}\log\;(q_{i})=-\log\;(q_{t})$ with $p_{i}=1$ for i=t and zero otherwise. Our formulation can thus be considered as a natural generalization of cross entropy loss commonly used in deterministic artificial neural networks. The physical significance of Eq. 4 is that by minimizing L(θ) the spiking neural network can be trained to make correct predictions while simultaneously minimizing trial-to-trial variability.

Although the general theoretical framework presented above provides a useful conceptual guide, question remains as how the spiking neural network under such probabilistic representation can be computed and trained. Direct evaluation of Eq. 2 is computationally infeasible at large scale and it is unclear how learning algorithms such as backpropagation can be implemented with respect to fluctuating neural activity. To resolve this problem, we employ a moment closure which parameterizes the probability distributions of neural spiking activity in terms of their first- and second-order statistical moments.

### A moment closure for gradient-based learning in spiking neural network

**Fig. 2.**
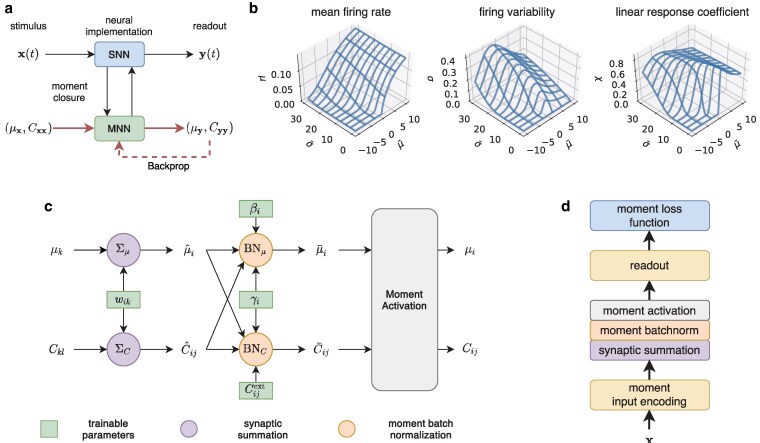
Gradient-based learning in spiking neural network through moment closure. a), Overall schematics. The spiking neural network model is first mapped to a corresponding moment neural network which can be trained with backpropagation; the trained weights are used to recover the original spiking neural network. b), Components of the moment activation function including mean firing rate *μ*, firing variability *σ*, and the linear response coefficient *χ*, each of which is a function of the input current mean $\bar{\mu }$ and variability $\bar{\sigma }$. In conventional analog and digital computing, such noise coupling is considered detrimental to information carried within the signal. In contrast, stochastic computing actively exploits correlated variability as a part of the computational processes. c), Computational graph of a single feedforward layer of the moment neural network, featuring synaptic summation, moment batch normalization and moment activation. d), Illustration of a trainable moment neural network with a feedforward architecture consisting of an input layer, arbitrary number of hidden layers, a readout layer, and a moment loss function.

The moment closure characterizes fluctuating neural spike count with its first- and second-order statistical moments, that is, the mean firing rate *μ* and the firing co-variability *C*. Through a diffusion formalism (77), we can derive on a mathematically rigorous ground the mapping from the statistical moments of the presynaptic neural activity to that of the synaptic current, and from the synaptic current to the postsynaptic neural activity. This leads to a class of neural network models known as the *moment neural network* (MNN) which faithfully captures spike count variability up to second-order statistical moments (65, 66). This can be considered as a minimalistic yet rich description of stochastic neural dynamics characterizing all pairwise neural interactions. The moment closure essentially provides a finite-dimensional parameterization of joint probability distributions of neural spiking activity through which gradient-based learning can be performed. The network parameters trained this way can then be used directly to recover the spiking neural network without fining tuning of free parameters. An overall schematic illustrating this concept is shown in Fig. 2a.

In the following, we present the main results of the moment closure for the leaky integrate-and-fire (LIF) spiking neuron model, though the general approach is applicable to any type of spiking neuron model (69), and construct the basic building blocks of the MNN, namely, synaptic summation, moment activation, moment batch normalization, and moment loss functions. The main results are summarized in Table 1. In the following, we explain how the moment closure for each of these building blocks are derived, with a great emphasis placed on the correspondence between SNN, MNN, and ANN. Our analysis offers new insights to the connections between spiking and continuous-valued neural network models through the lens of stochastic neural computing. Full details of the derivation are presented in Methods section [see Eqs. 9–15].

**Table 1.** Relationship between spiking neural network (SNN), rate-based artificial neural network (ANN), and moment neural network (MNN) through the lens of stochastic neural computing.

	SNN	Rate-based ANN	MNN
State variables	Spike count:	Mean firing rate:	Mean firing rate and firing covariability:
	$n(\Delta t)=\int_{0}^{\Delta t}S(t^{\prime })dt^{\prime }$	$\mu =\lim_{\Delta t\to \infty }\frac{E[n]}{\Delta t}$	$\mu =\lim_{\Delta t\to \infty }\frac{E[n]}{\Delta t}$
			$C=\lim_{\Delta t\to \infty }\frac{\mathrm{Cov}[n,n]}{\Delta t}$
Synaptic summation	Linear transformation of spike train by synaptic weights:	Linear transformation of mean firing rate with synaptic weights:	Linear/bilinear transformation of mean firing rate/covariance by synaptic weights:
	$I(t)=WS(t)+I_{\mathrm{ext}}$	$\hat{\mu }=W\mu +\mu_{\mathrm{ext}}$	$\hat{\mu }=W\mu +\mu_{\mathrm{ext}}$
			$\hat{C}=WCW^{T}+C_{\mathrm{ext}}$
Normalization	Normalizing postsynaptic current:	Batchnorm:	Moment batchnorm:
	$I\leftarrow \frac{I-E[I]}{\sqrt{\mathrm{Var}[I]}}\gamma +\beta$	$\bar{\mu }=\mathrm{BN}(\hat{\mu })$	$\left(\bar{\mu },\bar{C})=\mathrm{MBN}(\hat{\mu },\hat{C}\right)$
Neuronal activation	Spiking dynamics:	Pointwise activations (e.g. sigmoid, ReLU):	Moment activation:
	$\frac{dV}{dt}=-LV+I,$	$\mu =\phi (\bar{\mu })$	$\left(\mu ,C)=\phi (\bar{\mu },\bar{C}\right)$
	$V\leftarrow V_{\mathrm{res}}$ when $V>V_{\mathrm{th}}$		
Loss functions	Negative log-likelihood:	Mean squared error:	Moment mean squared error:
	−logp(y|x,θ)	$\|\mu -y^{*}{\|}^{2}$	$\left(\mu -y^{*}{)}^{T}C^{-1}(\mu -y^{*})\Delta t+\log\;\text{det}(\frac{2\pi }{\Delta t}C\right)$
	Probability of correct predictions:	Cross-entropy:	Moment cross-entropy:
	$\int p(y|x,\theta )1_{D_{i}}(y)dy$	$-\log\;\sigma_{t}(\mu ;\beta )$	$-\log\;\sum_{n=1}^{N}\sigma_{t}(\frac{1}{\sqrt{\Delta t}}Lz^{n}+\mu ;\beta )$

The MNN systematically generalizes conventional deep learning architectures to second-order and serves as a bridge between spiking and continuous-valued neural networks.

#### Synaptic summation

Synaptic summation for spiking neurons involves linearly transforming the presynaptic spike trains S(t) by the synaptic weight *W* to obtain the postsynaptic current I(t). Its moment closure then corresponds to a mapping from the statistical moments of presynaptic neuron spikes to that of the postsynaptic currents. For the mean mapping, the synaptic summation works similarly as in standard rate models by calculating the synaptic current mean as a weighted sum of the presynaptic mean firing rate [Eq. 11]. Unlike rate models, however, the same synaptic weights are also used to transform the second-order moments [Eq. 12], resulting in correlated synaptic currents even if the input spikes are uncorrelated.

#### Moment activation

When a spiking neuron receives fluctuating synaptic currents, it generates irregular spiking activity. There then exists a mapping from the statistical moments of the synaptic current to that of the spiking activity of postsynaptic neurons. An important feature of this moment mapping is that the mean and covariances are nonlinearly coupled, as found in cortical neurons in the brain (5). This moment mapping, termed the moment activation, can be derived analytically from spiking neuron models via a combination of mathematical techniques including diffusion approximation and linear response theory (5, 65, 66). The moment activation for the leaky integrate-and-fire neuron model is given by Eqs. 13–15. Figure 2b illustrates the three components of the moment activation, namely, the mean firing rate, the firing variability, and the linear response coefficient. An efficient numerical algorithm with custom gradients is used for implementing the moment activation (74).

#### Moment batch normalization

In deep networks, when the inputs are sufficiently strong (or weak), it may cause the saturation (or vanishing) of the moment activation function and subsequently the failure of gradient propagation. For conventional rate-based activation functions such as sigmoid functions, this vanishing-gradient problem is effectively alleviated through batch normalization (78). Here, we propose a generalized batch normalization incorporating second-order moments, referred to as the moment batch normalization [see Eqs. 20 and 21 in Methods section]. A key property of the moment batch normalization is that a common normalization factor is shared between the mean and variance of the synaptic current. Remarkably, it can be shown that the MBN is equivalent to a scaling and biasing over the postsynaptic current in the spiking neural network. As a result, the scaling and bias parameters can be re-absorbed into the synaptic weights and external input currents respectively after training is complete, thereby preserving the structure of the original spiking neural network [see Eqs. 24 and 25].

#### Moment loss functions

To enable gradient-based learning, we also need to specify an appropriate loss function. The general framework of stochastic neural computing offers a principled approach to deriving loss functions incorporating second-order moments. For regression problems, we derive the moment mean squared error [MMSE, Eq. 22] based on the principle of maximum likelihood [Eq. 3]. This loss function simultaneously minimizes the difference between the output mean *μ* and the target $y^{*}$ (systematic error) in the first term as well as the output covariance (random error) in the second term. The readout time Δt controls the trade-off between accuracy and precision, that is, a smaller Δt prioritizes reducing the random error more than the systematic error and vice versa. Interestingly, Eq. 22 can be interpreted as a form of free energy, such that the first and the second terms correspond to the energy and the entropy of the system, respectively. The standard mean-squared error (MSE) loss is a special case of Eq. 22 for when C=I.

For classification problems, a commonly used loss function in conventional ANNs is the softmax cross entropy, in which model predictions are formally assigned to a probability via the softmax function, often with analogy drawn to the Boltzmann distribution from statistical physics. However, whether the output of the softmax function can truly be interpreted as probability is questionable since there are no underlying random processes involved. In contrast, the SNC framework allows us to define classification accuracy in the native language of probabilities, that is, trial-wise probability of correct predictions [Eq. 4], whose moment closure leads to the moment cross-entropy (MCE) loss [Eq. 23]. The standard cross-entropy loss used in ANNs turns out to be a special case of the MCE when the readout time is unlimited, that is, when Δt→∞. Remarkably, our analysis reveals the true nature of the softmax function as an approximation to the indicator function appearing in Eq. 4, rather than representing some probability distribution as commonly misinterpreted.

Given these basic building blocks of MNN, we can now assemble a single feedforward layer of MNN by connecting synaptic summation, moment batch normalization, and moment activation, as illustrated in Fig. 2c. By mapping the mean firing rate and firing covariability of the presynaptic population to that of the postsynaptic population, this feedforward layer essentially implements one step of stochastic neural computing in Eq. 1 which maps $p(n_{k-1}|x)$ to $p(n_{k}|x)$. Multiple hidden layers can be stacked together to form a network of arbitrary depth, effectively implementing the chain of probabilistic computation in Eq. 2. To enable end-to-end learning, it is also necessary to specify an appropriate moment representation for the input and the readout for making inference. Here we assume independent Poisson input encoding, though our approach allows for any covariance structures within the input. For inference, we apply a linear readout on the last hidden layer [Eqs. 18 and 19], which is similar to synaptic summation. Figure 2d shows an example of a complete feedforward MNN consisting of a Poisson-encoded input layer, a hidden layer, a linear readout, and a moment loss function.

In summary, the moment closure presented above leads to a new class of neural network model, the moment neural network, which is derived from SNN on a mathematically rigorous ground and at the same time naturally generalizes ANNs to second-order. The MNN serves as a conceptual link between ANN and SNN which has been missing in the literature, and provides a unifying perspective of the relationship between spiking and continuous-valued neural network models through the lens of stochastic neural computing.

### Stochastic neural computing with correlated variability

Having developed the basic building blocks of MNN, we now demonstrate our learning framework for SNC with a classification task. For illustrative purposes, we consider a fully connected, feedforward MNN for implementing supervised learning on the MNIST datasest (79) consisting of images of hand-written digits. A single hidden layer and a Poisson-rate input encoding scheme are used. See Methods section for details of model set-up.

**Fig. 3.**
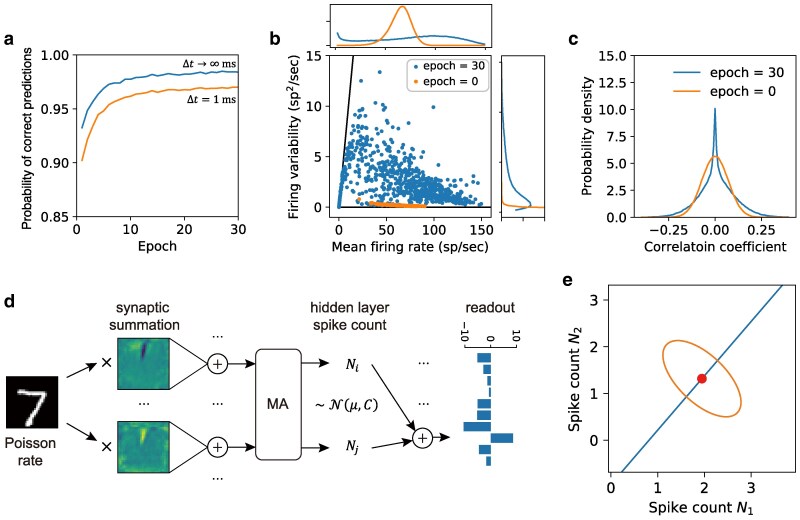
Moment neural network learning classification task while simultaneously minimizing uncertainty. a) The probability of correct prediction averaged over all samples of the validation set during training for unlimited and limited readout time Δt during inference; the latter takes into account of trial-to-trial variability. For unlimited readout time (Δt→∞) during inference, the accuracy reaches 98.45% at the end of the epochs, comparable to the performance of rate-based artificial neural networks. b) Diverse firing variability of hidden layer neurons in response to the input image shown in e), exhibiting both mean-dominant (Fano factor close to zero) and fluctuation-dominant (Fano factor close to one, solid line) activity. Insets: probability densities. c) Spike count correlation of the hidden layer neurons exhibit weak correlation whose distribution shows a slower decaying tail after training. d) Illustration of nontrivial roles played by correlated variability of a specific pair of hidden layer neurons. An input image represented by independent Poisson spike trains undergoes synaptic summation with anti-correlated weights, leading to anti-correlated neural activity. The final readout is linearly decoded from the hidden layer spike counts. e) The mean (dot) and covariance (ellipse) of the spike count of those two neurons over a readout time Δt=100 ms. In this example, the principal axis of the covariance is orthogonal to the direction of the readout weights (solid line) with respect to the target class, leading to a reduction in the readout variance and simultaneously to an increase in the readout mean.

Figure 3a shows the classification accuracy, measured as the probability of correct prediction [$q_{t}$ in Eq. 4] averaged across all images in the validation set, increases with training epochs. When the readout time is infinite [Δt→∞ in Eq. 4], this simply reflects the fraction of correctly classified samples like in a rate-based artificial neural network. In contrast to rate models, however, the MNN can also express uncertainty (trial-to-trial variability) when the readout time is finite, as reflected by a lower probability of correct prediction. Note that as Δt increases, the probability of correct prediction at finite readout time converges to the theoretical limit. As we will show in a later section, this convergence is exponentially fast with a time scale directly related to the readout uncertainty.

In addition, the hidden layer exhibits diverse firing variability consistent with cortical neurons (4, 80, 81). Figure 3b shows the neural response to a typical sample image, with each point corresponding to a neuron. The mean firing rate and firing variability of the hidden layer neuron cover a broad range of values, from fluctuation-dominant activity (closer to Fano factor of one, solid line) to mean-dominant activity (closer to Fano factor of zero, *x*-axis). In contrast, a network with random initialization before training has narrowly distributed firing variability. The pairwise correlations of the hidden neurons are also weakly correlated, with both positive and negative values centered around the origin [Fig. 3(c)]. This result is consistent with that observed in cortical neurons (8, 81), and also satisfies the assumptions behind the linear response analysis used to derive the correlation mapping in Eq. 15. Interestingly, we find that the distribution of the correlation coefficients exhibits a longer tail after training.

To provide an intuitive understanding about the role played by correlated neural variability, we now focus on a specific pair of neurons in the hidden layer and trace the computational steps involved in producing $y_{t}$, the readout component corresponding to the target class. As shown in Fig. 3d, an input image encoded by independent Poisson spikes with $\mu_{0}=\sigma_{0}^{2}$ first undergoes synaptic summation to produce correlated synaptic currents, which in turn elicit neural responses $\left(\mu_{1},C_{1}\right)$ in the hidden layer. For the specific pair of neurons shown, the synaptic weights have opposite patterns, resulting in negatively correlated neural responses. Synaptic weights to other representative neurons are shown in Fig. S3 in the Supplementary information. Figure 3e illustrates the joint distribution of spike count (Δt=100 ms) for this neuronal pair, with their mean firing rate marked by the dot and their covariance highlighted by the ellipse. Remarkably, the principal axis of the covariance, in this 2D projection, is orthogonal to the line representing the readout weights from these two neurons to the target class [solid line in Fig. 3e]. As a result, the readout effectively projects the spike count distribution in the hidden layer along its principal axis, leading to reduced uncertainty in $y_{t}$.

A more detailed analysis of neural coding properties of hidden neurons (Fig. S1) reveal that hidden neurons can be broadly categorized as accuracy or precision contributors and the neural code is highly distributed such that each neuron can play multiple roles across different input classes. For some neurons, even when they do not contribute to inference accuracy in their mean firing rate, the co-fluctuations between them could nonetheless reduce uncertainty. This aligns with experimental studies which have identified an association between behavioral uncertainty and decoding error (82), and between neural pairwise correlations and behavioral uncertainty (32). Future experimental studies could specifically look into the existence of such “precision contributors” among cortical neurons.

To demonstrate the general applicability and scalability of our method, we conduct additional experiments on the Fashion-MNIST and CIFAR-10 datasets with deeper networks (see Supplementary information for model settings). The prediction accuracies of these models on the test sets are summarized in Table 2. We find that for all tasks, the performance of the MNN is comparable to its ANN counterpart under the same network structure. Using the trained weights of the MNNs, we reconstruct the corresponding SNNs (we will come to this point in the next section) and find that their performances are remarkably close to those of the MNNs, considering that no additional parameters are introduced during and after training. We have not included larger datasets like CIFAR-100 or ImageNet as it would require an extension of the MNN to convolutional architectures. This extension is conceptually straightforward, as it would require replacing the matrix multiplication in Eq. 12 with a double-sided convolution. The main challenge is technical as there is currently no available convolution kernel for covariance and developing efficient code infrastructure is a direction of future works.

**Table 2.** Comparison of model performance on MNIST, Fashion-MNIST and CIFAR-10 datasets.

Dataset	Model structure	Model type	Loss function	Accuracy (%)	SNN’s accuracy (%)
MNIST	784-1000-10	MNN	MCE (Δt=1)	98.58 ± 0.09	98.60 ± 0.02
			MCE (Δt=10)	98.70 ± 0.10	98.70 ± 0.03
			CE	98.68 ± 0.03	98.69 ± 0.03
		ANN	CE	98.85 ± 0.07	–
Fashion-MNIST	784-1000-10	MNN	MCE (Δt=1)	86.72 ± 0.26	86.71 ± 0.07
			MCE (Δt=10)	87.13 ± 0.38	87.14 ± 0.08
			CE	87.25 ± 0.32	87.20 ± 0.08
		ANN	CE	88.44± 0.18	–
CIFAR-10	3072-1000-1000-1000-10	MNN	MCE (Δt=1)	55.24 ± 0.38	49.79 ± 0.21
			MCE (Δt=10)	54.95 ± 1.18	51.12 ± 0.22
			CE	55.17 ± 1.40	52.53 ± 0.23
		ANN	CE	60.36 ± 0.75	–

The reported accuracies reflect the mean and standard deviations across 5 training trials on the test dataset. Here, Δt is the hyperparameter used in the MCE loss during training. For the SNN, the reported accuracies reflect the mean and standard deviations across 100 inference trials.

### Reconstructing spiking neural network with zero free parameter

Because the MNN is analytically derived from the spiking neural network (SNN) model, recovering the SNN from a trained MNN is straightforward. No further post-training optimization or fining tuning is required. First, an input image is encoded into independent Poisson spike trains, which then undergo synaptic summation according to Eq. 7. The synaptic weights and the external currents $I_{i}^{\mathrm{ext}}$ are recovered by absorbing the moment batch normalization into the summation layer of the trained MNN according to Eq. 25. Finally, the readout y(Δt) is calculated from the spike count n(Δt) over a time window of duration Δt according to Eq. 8. It becomes evident that the readout y(Δt) follows a distribution p(y|x;θ) with mean μ(θ) and covariance $\frac{1}{\Delta t}C(\theta )$ as output by the MNN. The class corresponding to the largest entry in the readout y(Δt) is then taken as the class prediction.

**Fig. 4.**
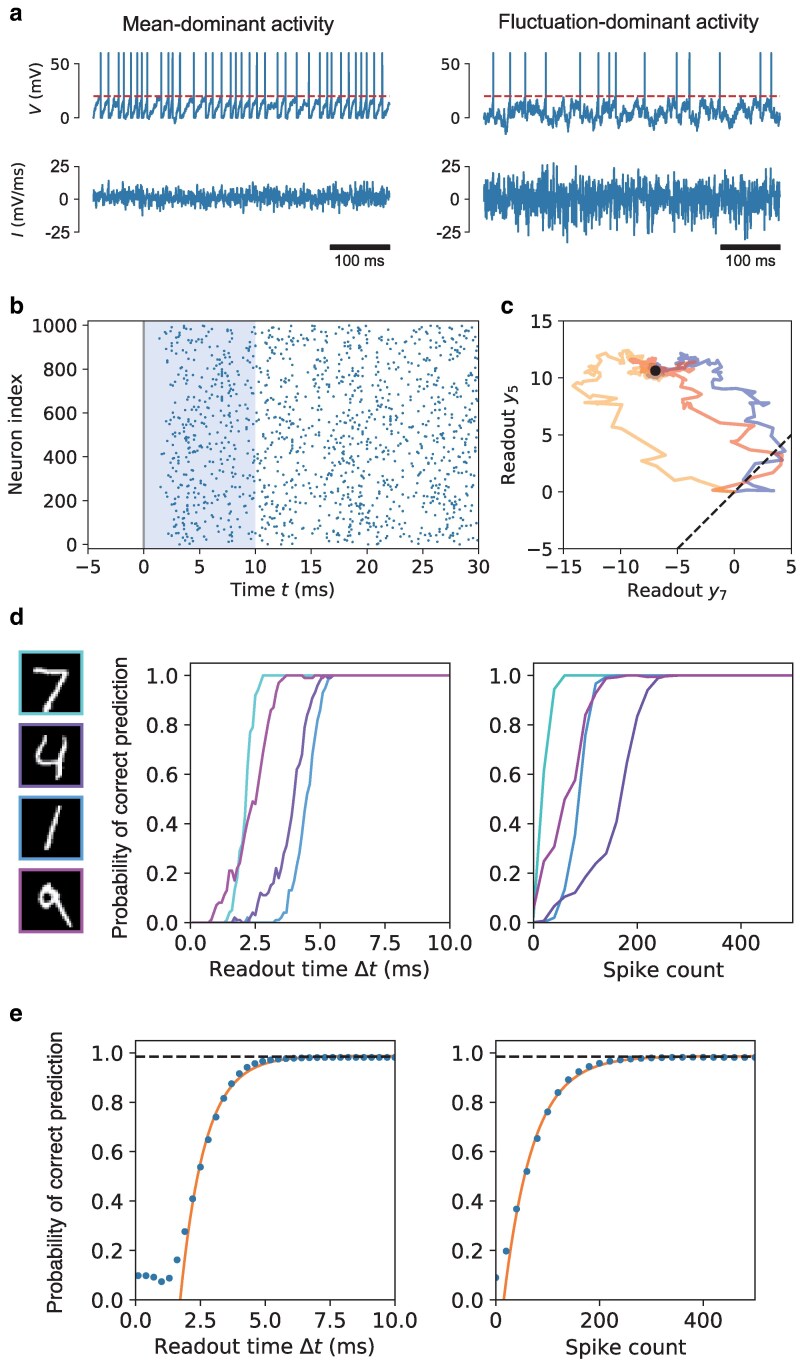
Temporal dynamics of stochastic neural computing in spiking neural network. a) Membrane potential (upper panel) and synaptic current (lower panel) of two typical hidden layer neurons during one trial of stimulus presentation, each exhibiting mean-dominant (left panel) and fluctuation-dominant (right panel) activity. Dashed line indicates firing threshold. b) Raster plot of typical spiking activity of hidden layer neurons during one trial of stimulus presentation. Solid line indicates stimulus onset at t=0 ms; shaded region indicates the readout time window Δt. The membrane potential of all neurons are initialized to zero at Δt=0 ms. c), Two dimensional projection of the readout trajectory *y* over time for three trials using the same stimulus. The vertical axis is the readout component corresponding to the correct class. Dot indicates theoretical limit of the readout mean as Δt→∞; dashed line indicates the decision boundary. d), Probability of correct prediction for a number of input images (left panel) as a function of readout time and population spike count in the hidden layer. Each curve is calculated from 100 trials with the same stimulus. e), Probability of correct prediction averaged over all images of the validation set converges exponentially with the readout time (left panel) as well as the population spike count in the hidden layer (right panel). Dashed lines indicate the theoretical limit of 0.985 as predicted by the MNN; solid lines represent exponential fits.

As consistent with the MNN, the recovered SNN exhibits both mean-dominant and fluctuation-dominant spiking activity as shown in Fig. 4a. For a typical neuron with mean-dominant activity, the synaptic current it receives has a positive mean and weak temporal fluctuations. As a result, the sub-threshold membrane potential of the neuron consistently ramps up over time, resulting in spike emission at relatively regular intervals. In contrast, a neuron with fluctuation-dominant activity is largely driven by a synaptic current with large fluctuations even though its mean is close to zero, resulting in spike emission at highly variable intervals. Such diverse firing variability is a key feature of SNC, even if the neuronal model itself is deterministic. The spike raster plot of hidden layer neurons in the SNN in response to an input image [the same as in Fig. 3d] is shown in Fig. 4b. We find that the MNN accurately captures the distribution of neural activity in the reconstructed SNN (see Fig. S2).

To reveal the temporal dynamics of the readout, we show in Fig. 4c a 2D projection of the readout trajectories y(Δt) in response to the same image over different trials. When Δt is small, the readouts from individual trials are scattered over a wide area, corresponding to a larger trial-to-trial variability. As more spikes are accumulated with increasing Δt, the readout trajectory in a single trial also fluctuates over time and eventually converges toward the readout mean *μ* [marked with the dot in Fig. 4c] as predicted by the MNN. Since the magnitude of the fluctuations in the readout tends to decrease over time, this may potentially provide a way for the brain to infer confidence during a single trial and potentially an early stopping criterion for decision-making.

To further quantify how task performance depends on readout time, we simulate the SNN over 100 trials for each image in the validation set of MNIST, and calculate the probability of correct prediction [$q_{t}$ in Eq. 4] for different input images as the readout time Δt increases. As can be seen from the result for four randomly picked images shown in Fig. 4d, $q_{t}$ increases with the readout time rapidly and eventually reaches one within around 5 ms, with some images require less time than others. A similar pattern is found for $q_{t}$ when plotted as a function of spike count (measured by binning individual trials based on the population spike count of hidden layer neurons) which directly reflects the energy cost.

When averaged over all images, the probability of correct prediction reveals an exponential convergence toward the theoretical limit of 0.9845 as predicted by the MNN with a short time constant of τ=0.95 ms (left panel in Fig. 4e). A short burn-in time of around 1 ms is due to the membrane potential being initialized to zero. This rapid convergence results in short decision latency, with an average probability of correct prediction of 0.980 obtained in 6.6 ms. This is largely due to that the moment cross entropy explicitly takes into account of trial-to-trial variability for finite readout time, so that the neural network learns to improve the rate of convergence without requiring knowledge of precise spike timing. A similar exponential convergence for $q_{t}$ averaged over all images is found with respect to the spike count in the hidden layer, with a decay constant of around 50 spikes. An average probability of correct prediction of 0.980 can be achieved with 300 spikes in the hidden layer (985 spikes across the entire network if including the input spikes). This exceptional energy efficiency is largely due to that a large proportion of the neural population in our model is fluctuation-dominant [Fig. 3(a)], with an average firing rate of 50 sp/s per neuron across the hidden layer and 133 sp/s per neuron in the input layer.

To gain theoretical insights about this exponential convergence, consider a binary classification problem involving a scalar readout $y=\frac{1}{\Delta t}\sum_{i}w_{i}n_{i}(\Delta t)$. Suppose that the correct class corresponds to readout values above a decision boundary *θ* and that the readout follows a normal distribution with mean μ>θ and variance $\sigma^{2}/\Delta t$. Then, the probability of correct prediction is simply $q(\Delta t)=\frac{1}{2}[1+\mathrm{erf}(\sqrt{\lambda \Delta t})]$, where $\lambda =\frac{(\mu -\theta {)}^{2}}{2\sigma^{2}}$. Its convergence property for large Δt is revealed by its asymptotic expansion


(5) $$q(\Delta t)∼1-\frac{1}{\sqrt{4\pi \lambda \Delta t}}\exp\;\left(-\lambda \Delta t\right).$$

This analysis shows that as Δt increases, the probability of correct prediction *q* converges exponentially toward one, and as it turns out *λ* plays the role of convergence rate. As *λ* is inversely proportional to the readout variance $\sigma^{2}$, our analysis provides a quantitative explanation why the inference speed can be improved by minimizing $\sigma^{2}$. For higher dimensions and decision boundaries with complex shapes, the probability of correct prediction does not have simple expressions but the general principle still applies.

These results are consistent with biological studies on decision making which suggest that reaction time can be predicted by the distance to a classifier decision boundary through neural activation space (83). However, our results also indicate that mean firing rate and neural correlation may contribute to reaction time in different ways. Future experiments could investigate the association between neural correlations and reaction time in human and animals. For a quantitative comparison to previous methods for training SNN, see Table S1.

### Benchmark results on neuromorphic hardware

To further demonstrate our method, we implement the SNN trained through moment closure on Intel’s Loihi neuromorphic chip and provide benchmark results on accuracy, energy cost, and latency. For this purpose, we train MNNs on the MNIST dataset with one hidden layer and then transfer the trained parameters to a discrete-time SNN. The stimulus transduction factor is set to α=0.1 sp/ms for encoding input samples. See Supplementary information for details of SNN implementation on Loihi.

**Fig. 5.**
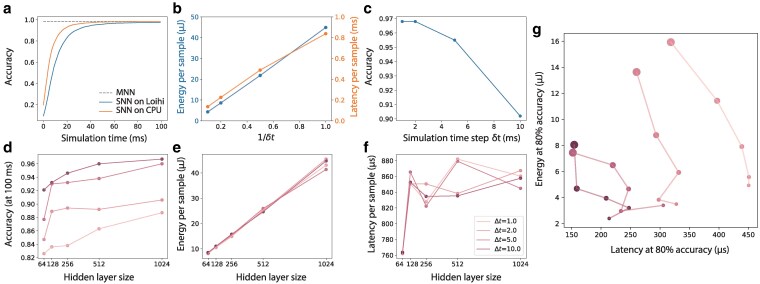
Performance of SNN deployed on neuromorphic chip. a) Classification accuracy increases with simulation time steps and converges to the theoretical limit predicted by the MNN. Compared to single-precision floating-point simulation on CPU, the simulation on Loihi has a small amount of accuracy loss caused by weight quantization. b) The classification accuracy over time of each model. c) The classification accuracy (at 100 ms of simulation time) for varying hidden layer size and Δt used in loss for training. d) The average energy cost per sample (at 100 ms of simulation time) for varying hidden layer sizes and Δt. e) The average latency (at 100 ms of simulation time) for varying hidden layer size and Δt. f) Energy-latency diagram (at 80% accuracy) revealing a trade-off between energy cost and latency. The dot size corresponds to hidden layer size and the color corresponds to the value of Δt used during training.

We first verify the consistency in prediction accuracy between the SNN on Loihi and those on a CPU. As shown in Fig. 5a, the prediction accuracy of the SNN on Loihi improves over time, approaching the theoretical limit of the MNN (indicated by the dashed line). Although the Loihi implementation has a minor accuracy reduction due to its 9-bit weight quantization when compared to the CPU’s single-precision floating-point implementation, the outcomes are remarkable given the absence of fine-tuning of free parameters post-training.

On neuromorphic hardware, a larger time step δt is often beneficial to lower both energy usage and delay. As illustrated in Fig. 5b, energy consumption and delay are approximately inversely proportional to δt given a fixed simulation duration (T=100 ms). As time discretization becomes coarser the accuracy diminishes (Fig. 5c). This occurs because the model parameters are optimized for a continuous LIF model instead of its discrete variant. This accuracy decline is minimal when δt is significantly less than the membrane time constant, 1/L=20 ms. To balance accuracy with energy efficiency, we choose δt=1 ms for subsequent analyses. To overcome this limitation, the moment closure method could be adapted in the future to directly develop moment mapping for the discrete SNN model.

In the following, we train MNNs with varying hidden layer sizes and Δt in the MCE loss to analyze how they affect SNNs’ accuracy, energy consumption, and delay when deployed on Loihi. Figure 5d shows that a larger hidden layer generally boosts accuracy, though the improvements diminish beyond the 512 mark. Accuracy also benefits from increasing the readout time Δt in the MCE loss during training. Meanwhile, Fig. 5e indicates that the dynamic energy per sample increases linearly with hidden layer size, remaining mostly unaffected by Δt. Lastly, Fig. 5f shows that latency per sample does not exhibit a clear pattern with varying network sizes or Δt.

The results above show that accuracy, energy consumption, and delay are intricately dependent on model hyperparameters, hinting at potential strategies for balancing the trade-offs between these factors. We are specifically interested in optimizing the energy-delay product for a particular accuracy level. To achieve this, we determine the inference time at 80% accuracy and record the corresponding energy and delay. This results in an energy-delay diagram shown in Fig. 5g. Each curve depicts the energy-delay profile for a given Δt as the hidden layer size changes. Our findings indicate that a larger hidden layer generally reduces latency but increases energy consumption, while an increase in Δt during training improves energy and reduces latency.

## Discussion

In this work, we have presented a theory of stochastic neural computing (SNC) and a way to implement it in spiking neural networks (SNN) through moment closure. The proposed method enables gradient-based learning in SNN in the noise-driven regime, where direct training using BPTT or ANN-to-SNN conversion would face challenges. It leads to a new class of deep learning architecture referred to as the moment neural network (MNN) which naturally generalizes standard deep learning architectures to second-order statistical moments and provides a conceptual link between SNN and continuous-valued ANN, and between spike-time coding and rate coding. Although the example presented here only considers a feedforward architecture with generic weights, future works may apply moment closure to systematically generalize many of the known deep learning architectures, such as convolutional and recurrent neural networks, to second-order statistical moments.

Compared to existing methods to training SNN, the proposed method has a number of additional advantages. First, unlike ANN-to-SNN conversion methods, which require extensive post-training optimization (37–39, 41–43), the MNN represents a tight approximation to SNN. As a consequence, once the MNN is trained, we can obtain the corresponding SNN for free, with no further fine tuning of hyperparameters needed. Second, compared to direct training of SNN, the MNN has less temporal complexity as it does not require the fine temporal resolution needed for simulating the membrane potential dynamics. In the case of a feedforward network, this is particularly advantageous because it avoids BPTT which is more expensive and harder to train than BP. The trade-off is increased spatial complexity for representing the covariance matrix which can become a limiting factor when network size becomes large. Detailed comparisons on the computational costs for simulating SNN and MNN are reported elsewhere (74). Third, once an MNN is trained, we also simultaneously obtain an analytical model for explaining the computational processes involving mean and covariance of neural activity. This provides a degree of analytical tractability and interpretability that is unavailable using other methods.

Moment closure as a general approach provides a finite-dimensional parameterization of a joint probability distribution using statistical moments and is an invaluable tool for studying stochastic processes in physical systems. In neuroscience, moment closure has also been used for modeling stochastic neural dynamics and coding properties of neural circuits (5, 31, 64, 71, 71–73). However, conventional modeling studies often rely on handcrafted approaches with prior assumptions regarding the form of neural code or simplifications for facilitating theoretical analysis (15, 20). In contrast, our method enables end-to-end learning of arbitrary probabilistic computation tasks and direct manipulations of correlated neural variability in a task-driven way. Therefore, the proposed method could serve as a useful tool for studying probabilistic neural computation and the functional role of correlated neural variability in the brain. For instance, the method developed in this works has been used to demonstrate how a single layer of spiking neurons can extract perceptual information hidden within the correlation of noisy input (84).

Although the tasks considered in this work are deterministic in nature, the learning framework based on moment closure can also be applied to probabilistic tasks. Indeed, there is a growing trend of using deep learning approaches to implement probabilistic computation in neural networks (85, 86). Reverse engineering the trained network could potentially provide valuable insights to probabilistic neural computation otherwise unattainable through handcrafted models for solving specifically curated problems.

The SNC theory places a strong emphasis on uncertainty representation through the stochastic processes of neural spike trains, through which signal and noise are processed concurrently rather than through different channels such as in a variational auto-encoder. An interesting idea is the use of MNN for calculating the mean and covariance that appear in various machine learning models. When mapped back to SNN, probability distributions are processed through spike-based sampling so that mean and covariance are implicitly processed using a single pool of neurons. A potential tradeoff is that the mean and covariance in MNN are propagated using shared synaptic weights, which implies less trainable parameters and less range of expression. Future works could test this idea in a range of machine learning models using the principle of SNC.

The approach developed in this paper also has broader implications to stochastic computing, which has been proposed as an alternative computing architecture for approximate computation with better error tolerance and energy efficiency (87, 88). However, designing stochastic computing circuits for arbitrary functions remains a challenge. Our method indicates a solution to this problem by training SNNs to implement spike-based SNC. The association between readout uncertainty and inference speed, as suggested by our SNC theory, also hints a way to design optimal stopping policies for fast inference in SNN (89). Combined with advances in neuromorphic hardware (85, 90, 91), the principle of SNC could lead to a future generation of brain-inspired computing architectures.

## Methods

### Leaky integrate-and-fire neuron model

The membrane potential dynamics of a leaky integrate-and-fire (LIF) neuron is described by


(6) $$\frac{dV_{i}}{dt}=-LV_{i}(t)+I_{i}(t),$$

where the sub-threshold membrane potential $V_{i}(t)$ of a neuron *i* is driven by the total synaptic current $I_{i}(t)$ and $L=0.05\;{\mathrm{ms}}^{-1}$ is the leak conductance. When the membrane potential $V_{i}(t)$ exceeds a threshold $V_{\mathrm{th}}=20\;\mathrm{mV}$ a spike is emitted, as represented with a Dirac delta function. Afterward, the membrane potential $V_{i}(t)$ is reset to the resting potential $V_{\mathrm{res}}=0$ mV, followed by a refractory period $T_{\mathrm{ref}}=5$ ms. The synaptic current takes the form


(7) $$I_{i}(t)=\sum_{\;j}w_{ij}S_{j}(t)+I_{i}^{\mathrm{ext}}(t),$$

where $S_{j}(t)=\sum_{k}\delta (t-t_{j}^{k})$ represents the spike train generated by presynaptic neurons.

A final output y is readout from the spike count n(Δt) of a population of spiking neurons over a time window of duration Δt as follows


(8) $$y_{i}(\Delta t)=\frac{1}{\Delta t}\sum_{\;j}w_{ij}n_{\;j}(\Delta t)+\beta_{i},$$

where $w_{ij}$ and $\beta_{i}$ are the weights and biases of the readout, respectively. One property of the readout is that its variance should decrease as the readout time window Δt increases.

### Moment closure for the leaky integrate-and-fire neuron model

The first- and second-order moments of spiking neural activity, which we refer to as the mean firing rate and firing co-variability, are defined as


(9) $$\mu_{i}=\lim_{\Delta t\to \infty }\frac{E[n_{i}(\Delta t)]}{\Delta t},$$

and


(10) $$C_{ij}=\lim_{\Delta t\to \infty }\frac{\mathrm{Cov}[n_{i}(\Delta t),n_{j}(\Delta t)]}{\Delta t},$$

respectively, where $n_{i}(\Delta t)$ is the spike count of neuron *i* over a time window Δt. The limits signify that we consider the statistics of the system after it has reached stationarity. For stationary processes, the trial-wise expectation of spike count over finite time windows does not depend on Δt, that is, $E[n_{i}(\Delta t)]=\mu_{i}\Delta t$ holds for all Δt. The covariance of spike count, however, always depend on the time window (as does Fano factor (92)), even if the system is stationary. In practice, we use $\mathrm{Cov}[n_{i},n_{j}]\approx C_{ij}\Delta t$ as an asymptotic approximation, which becomes increasingly accurate with larger Δt.

For the LIF neuron model [Eq. 6], the statistical moments of the synaptic current is equal to (65, 66)


$$\left\{ \begin{array}{cc} {\hat{\mu }}_{i}=\sum_{k}w_{ik}\mu_{k}+{\hat{\mu }}_{i}^{\mathrm{ext}}, & \left(11\right)\\ {\hat{C}}_{ij}=\sum_{kl}w_{ik}C_{kl}w_{\;jl}+{\hat{C}}_{ij}^{\mathrm{ext}}, & \left(12\right) \end{array}\right.$$

where $w_{ik}$ is the synaptic weight and ${\hat{\mu }}_{i}^{\mathrm{ext}}$ and ${\hat{C}}_{ij}^{\mathrm{ext}}$ are the mean and covariance of an external current, respectively. Note that from Eq. 12, it becomes evident that the synaptic current are correlated even if the presynaptic spike trains are not. Next, the first- and second-order moments of the synaptic current is mapped to that of the spiking activity of the postsynaptic neurons. For the LIF neuron model, this mapping can be obtained in closed form through a mathematical technique known as the diffusion approximation (65, 66) as


$$\left\{ \begin{array}{cc} \mu_{i}=\phi_{\mu }({\bar{\mu }}_{i},{\bar{\sigma }}_{i}), & \left(13\right)\\ \sigma_{i}=\phi_{\sigma }({\bar{\mu }}_{i},{\bar{\sigma }}_{i}), & \left(14\right)\\ \rho_{ij}=\chi ({\bar{\mu }}_{i},{\bar{\sigma }}_{i})\chi ({\bar{\mu }}_{j},{\bar{\sigma }}_{j}){\bar{\rho }}_{ij}, & \left(15\right) \end{array}\right.$$

where the correlation coefficient $\rho_{ij}$ is related to the covariance as $C_{ij}=\sigma_{i}\sigma_{j}\rho_{ij}$. In this paper, we refer this mapping given by Eqs. 13–15 as the moment activation.

The functions $\phi_{\mu }$ and $\phi_{\sigma }$ together map the mean and variance of the input current to that of the output spikes according to (65, 66)


$$\phi \;:\;(\bar{\mu },{\bar{\sigma }}^{2})\mapsto (\mu ,\sigma^{2}),\left\{ \begin{array}{cc} \mu =\frac{1}{T_{\mathrm{ref}}+\frac{2}{L}\int_{I_{\mathrm{lb}}}^{I_{\mathrm{ub}}}g(x)dx}, & \left(16\right)\\ \sigma^{2}=\frac{8}{L^{2}}\mu^{3}\int_{I_{\mathrm{lb}}}^{I_{\mathrm{ub}}}h(x)dx, & \left(17\right) \end{array}\right.$$

where $T_{\mathrm{ref}}$ is the refractory period with integration bounds $I_{\mathrm{ub}}(\bar{\mu },\bar{\sigma })=\frac{V_{\mathrm{th}}L-\bar{\mu }}{\sqrt{L}\bar{\sigma }}$ and $I_{\mathrm{lb}}(\bar{\mu },\bar{\sigma })=\frac{V_{\mathrm{res}}L-\bar{\mu }}{\sqrt{L}\bar{\sigma }}$. The constant parameters *L*, $V_{\mathrm{res}}$, and $V_{\mathrm{th}}$ are identical to those in the LIF neuron model in Eq. 6). The pair of Dawson-like functions g(x) and h(x) appearing in Eqs. 16 and 17 are $g(x)=e^{x^{2}}\int_{-\infty }^{x}e^{-u^{2}}du$ and $h(x)=e^{x^{2}}\int_{-\infty }^{x}e^{-u^{2}}[g(u){]}^{2}du$. The function *χ*, which we refer to as the linear perturbation coefficient, is equal to $\chi (\bar{\mu },\bar{\sigma })=\frac{\bar{\sigma }}{\sigma }\frac{\partial \mu }{\partial \bar{\mu }}$ and it is derived using a linear perturbation analysis around ${\bar{\rho }}_{ij}=0$ (66). This approximation is justified as pairwise correlations between neurons in the brain are typically weak (8). An efficient numerical algorithm is used for evaluating the moment activation and its gradients (74).

Finally, the moments of the readout $y_{i}$ [in Eq. 8] can be calculated as


$$\left\{ \begin{array}{cc} \;E[y_{i}]=\sum_{\;j}w_{ij}\mu_{j}+\beta_{i}, & \left(18\right)\\ \mathrm{Cov}[y_{i},y_{j}]=\frac{1}{\Delta t}\sum_{k,l}w_{ik}C_{kl}w_{\;jl}, & \left(19\right) \end{array}\right.$$

where $w_{ij}$ and $\beta_{i}$ are the weights and biases of the readout, respectively. Here, $\mu_{j}$ and $C_{ij}$ denote the mean firing rate and firing covariability as calculated by the moment activation [Eq. 15]. Note that in deriving Eq. 19, we have used the approximation $\mathrm{Cov}[n_{i},n_{j}]\approx C_{ij}\Delta t$. Unlike the readout mean, the readout covariance depends on the readout duration Δt, showing one of the striking differences between deterministic and stochastic neural computing.

An alternative approach considers firing probability in terms of the occupancy of refractory period (70, 93), which is equivalent to moment activation for very short time windows. The moment activation in the limit of large time window is advantageous in that it captures the full range of spike count Fano factors (or equivalently coefficient of variation of inter-spike intervals), as in Eqs. 16 and 17, which cannot be derived from a binary probability distribution. The second-order moments thus provide extra degrees of freedom to be involved in learning and inference. Future studies could combine these approaches to derive a moment activation that works for all Δt, rather than specific values or limits.

### Moment batch normalization

The moment batch normalization for the input mean ${\hat{\mu }}_{i}$ is


(20) $${\bar{\mu }}_{i}=\frac{{\hat{\mu }}_{i}-E[{\hat{\mu }}_{i}]}{\sqrt{\nu_{i}+\epsilon }}\gamma_{i}+\beta_{i},$$

where $E[{\hat{\mu }}_{i}]$ is the mean computed over samples within a mini-batch and $\nu_{i}$ is a normalization factor. The bias $\beta_{i}$ and scaling factor $\gamma_{i}$ are trainable parameters, similar to that in the standard batch normalization. The key difference from the standard batch normalization for firing rate model is the normalization factor which must accommodate the effect of input fluctuations. In this study, we propose the following form of normalization factor, $\nu_{i}=\mathrm{Var}[{\hat{\mu }}_{i}]+E[{\hat{\sigma }}_{i}^{2}]$, which involves the expectation of the input variance in addition to the variance of the input mean. In fact, by invoking the law of total variance, it can be shown that this particular choice of normalization factor can be interpreted as the variance of the total synaptic current $I_{i}(t)$ [Eq. 7] in the corresponding SNN, that is, $\mathrm{Var}[{\hat{\mu }}_{i}]+E[{\hat{\sigma }}_{i}^{2}]=\mathrm{Var}[I_{i}(t)]$, where the variance on the right-hand side is evaluated across the mini-batch as well as time. Note that the standard batch normalization used in rate-based ANN corresponds to the special case of Eq. 20 when the input current $I_{i}(t)$ is constant, that is, when ${\hat{\sigma }}_{i}^{2}=0$.

The moment batch normalization for the input covariance ${\hat{C}}_{ij}$ enforces the same normalization factor $\nu_{i}$ and trainable $\gamma_{i}$ as used in Eq. 20 but without centering. The shared normalization factor and trainable factor allow the moment batch normalization to be absorbed into the synaptic weights after training is complete, thereby preserving the link to the underlying spiking neural network. This leads to


(21) $${\bar{C}}_{ij}=\frac{{\hat{C}}_{ij}}{\sqrt{\left(\nu_{i}+\epsilon )(\nu_{j}+\epsilon \right)}}\gamma_{i}\gamma_{j}+C_{ij}^{\mathrm{ext}},$$

where $C_{ij}^{\mathrm{ext}}$ represents the covariance of an external input current. To ensure symmetry and positive semi-definiteness of the covariance matrix, we set $C^{\mathrm{ext}}=A^{T}A$ with the matrix *A* being a trainable parameter with the same size as $C^{\mathrm{ext}}$. Alternatively, for independent external input current, we set $C_{ij}^{\mathrm{ext}}=\delta_{ij}(\sigma_{i}^{\mathrm{ext}}{)}^{2}$, with $\sigma_{i}^{\mathrm{ext}}$ being trainable parameters. In practice, the computation of Eq. 21 can be quite cumbersome and one way to significantly simplify this step, with some reduced flexibility, is to consider the special case where the external input covariance $C_{ij}^{\mathrm{ext}}$ is zero. Under this scenario, we only need to apply batch normalization to the variance and pass directly the off-diagonal entries via the correlation coefficient, ${\bar{\rho }}_{ij}={\hat{\rho }}_{ij}$. Similar to the standard batch normalization, the input mean and input variance over minibatch are replaced by the running mean and running variance during the validation phase. A schematic diagram showing the moment batch normalization is shown in Fig. 2.

A practical benefit of the moment batch normalization is that it simplifies parameter initialization before training as we can initialize of the parameters $\left(\beta_{i},\gamma_{i},C_{ij}^{\mathrm{ext}}\right)$ to appropriate values so that the total postsynaptic current $\left({\bar{\mu }}_{i},{\bar{\sigma }}_{i}\right)$ is always within a desired working regime, regardless of the task or the input sample.

### Moment loss functions

Assuming a Gaussian-distributed readout and substituting its probability density


$$p(y|x;\theta )=\frac{1}{\sqrt{\text{det}(2\pi C/\Delta t)}}\exp\;\left[-\frac{1}{2}(y-\mu {)}^{T}C^{-1}(y-\mu )\Delta t\right]$$

into each of Eqs. 3 and 4 lead to the following objective functions expressed in terms of the second-order statistical moments of the readout. Here, we use *μ* and C/Δt to denote the readout mean E[y] and readout covariance Cov[y,y] in Eqs. 18 and 19.

For regression problems, the principle of maximum likelihood leads to


(22) $$L(\theta )=(\mu -y^{*}{)}^{T}C^{-1}(\mu -y^{*})\Delta t+\log\;\text{det}\left(\frac{2\pi }{\Delta t}C\right),$$

where $y^{*}$ represents the readout target and *t* represents matrix transpose. We refer this loss function as the moment mean-squared error (MMSE) loss. In practice, a small positive value (representing a constant external background noise) is added to the diagonal entries of *C* to avoid numerical instability during matrix inversion.

For classification problems, class prediction is obtained by taking the class label *i* corresponding to the largest entry of y. Since there is no simple analytical expression for the probability of correct predictions $q_{i}(\theta )=\int p(y|\theta )1_{D_{i}}(y)dy$ in high dimensions, we use a finite-sample approximation such that $q_{i}\approx \frac{1}{N}\sum_{n=1}^{N}1_{D_{i}}(y^{n})$, with $y^{n}$ being a multivariate normal random variable with mean *μ* and covariance $\frac{1}{\Delta t}C$. To generate the random samples, we perform Cholesky decomposition $C=LL^{T}$ to express $y^{n}$ as $y^{n}=Lz^{n}+\mu$, where $z^{n}$ is an uncorrelated unit normal random variable. Importantly, the Cholesky decomposition *L* is differentiable with respect to *C*, allowing for backpropagation to be implemented. Next, to solve the nondifferentiability of the indicator function, we approximate it with the soft-max function $1_{D_{i}}(y)\approx \sigma_{i}(y;\beta )=\exp\;(\beta y_{i})/\sum_{i}\exp\;(\beta y_{i})$, where *β* is a steepness parameter such that $\sigma_{i}(y;\beta )\to 1_{D_{i}}(y)$ as β→∞. Combining all these steps we obtain the following generalized cross-entropy loss


(23) $$H(\theta )\approx -\log\;\sum_{n=1}^{N}\sigma_{t}\left(\frac{1}{\sqrt{\Delta t}}Lz^{n}+\mu ;\beta \right),$$

which we refer to as the moment cross-entropy (MCE) loss (here *t* denotes target class). Note that the standard cross-entropy loss commonly used in deep learning corresponds to a special case of Eq. 23 when the readout time is unlimited, that is, when Δt→∞.

### Recovering synaptic weights in spiking neural network

The synaptic weights $w_{ij}$ and the moments of the external currents $I_{i}^{\mathrm{ext}}(t)$ in the SNN are recovered by absorbing the moment batch normalization into the summation layer of the trained MNN according to the formulae


(24) $$\mu_{i}^{\mathrm{ext}}=\beta_{i}-\frac{\gamma_{i}}{\sqrt{\nu_{i}+\epsilon }}E[{\hat{\mu }}_{i}],$$

(25) $$w_{ij}\leftarrow \frac{\gamma_{i}}{\sqrt{\nu_{i}+\epsilon }}w_{ij},$$

where $w_{ij}$ on the right-hand side is the synaptic weight of the summation layer in the trained MNN; the quantities $E[{\hat{\mu }}_{i}]$, $\nu_{i}=\mathrm{Var}[{\hat{\mu }}_{i}]+E[{\hat{\sigma }}_{i}^{2}]$, $\beta_{i}$ and $\gamma_{i}$ are the running mean, running variance, bias and scaling factor in the moment batch normalization [Eqs. 20 and 21]. The covariance of the external current $C_{ij}^{\mathrm{ext}}$ is the same as that in Eq. 21. The external current $I_{i}^{\mathrm{ext}}(t)$ to the spiking neural network can therefore be reconstructed as a Gaussian white noise with mean $\mu_{i}^{\mathrm{ext}}$ and covariance $C_{ij}^{\mathrm{ext}}$, and in turn be fed into the LIF neuron model in Eq. 7. No further post-training optimization or fining tuning is required during this reconstruction procedure.

### Model setups for training

We train the moment neural network on the MNIST dataset which contains 60,000 images for training and 10,000 images for validation. The model consists of an input layer, a hidden layer and a readout layer. For this task, the number of neurons is 784 for the input layer, 1,000 for the hidden layer, and 10 for the readout. For the input layer, a Poisson-rate encoding scheme is used such that neurons in the input layer emits independent Poisson spikes with rates proportional to the pixel intensity x, that is, $\mu_{0}=\sigma_{0}^{2}=\alpha x$, where *α* is the stimulus transduction factor set to be α=1 spikes per ms, and the correlation coefficient $\rho_{ij}=0$ for i≠j. The hidden layer involves synaptic summation [Eqs. 11 and 12], followed by moment batch normalization [Eqs. 20 and 21] and then by moment activation [Eqs. 13–15]. The readout mean *μ* and covariance $\frac{1}{\Delta t}C$ are calculated using Eqs. 11 and 12 where the readout time is set to be Δt=1 ms. The moment cross entropy loss [Eq. 23] is used to train the network. with the number of random samples set to be n=1,000 and the steepness parameter to β=1 during training. The model is implemented in Pytorch and trained with stochastic gradient descent (AdamW). Gradients are evaluated using Pytorch’s autograd functionality, except for the moment activation in which custom gradients for Eqs. 13–15 are used (see Supplementary information and also (74)). Also see Supplementary information for model setups for larger datasets.

## Supplementary Material

pgaf284_Supplementary_Data

## Data Availability

The code for simulating and training the moment neural network (MNN) model is available without restrictions on Github (https://github.com/BrainsoupFactory/moment-neural-network).

## Preprints

A preprint of this article is published at https://doi.org/10.48550/arXiv.2305.13982.
