# Supplementary material for: Learning and inference with correlated neural variability
Source: PNAS Nexus. 2025 Oct 10;4(10):pgaf284. doi: 10.1093/pnasnexus/pgaf284 (PMC12512019; doi:10.1093/pnasnexus/pgaf284)
Supplement: pgaf284_Supplementary_Data [file pgaf284_supplementary_data.pdf]

Supporting Information for

Learning and inference with correlated neural variability

Yang Qi, Zhichao Zhu, Yiming Wei, Lu Cao, Zhigang Wang, Jie Zhang, Wenlian Lu, Jianfeng Feng

Jianfeng Feng.
E-mail: jffeng@fudan.edu.cn

This PDF file includes:

Supporting text
Figs. S1 to S3
Table S1
SI References

Supporting Information Text

1. Quantitative comparison to previous methods for training SNN

In this section, We provide a quantitative comparison to previous methods for training SNN on the MNIST dataset under similar conditions as ours (table. S1). The following metrics are compared: accuracy for continuous-valued model (ANN and MNN) and the accuracy for SNN after convergence given sufficient time, inference time per sample, the total number of spikes per inference. The parameter settings including network size, membrane time constant, and firing threshold, and the techniques used are also commented. We find that while all methods summarized here can achieve similar performance in terms of the final accuracy, our method is superior in terms of inference time and the number of spikes (proportional to energy cost on neuromorphic hardware). Previously, best inference time and energy saving are achieved with post-training weight normalization. Here, the MNN provides an alternative approach with competitive performance without any post-training optimization or extra parameters during training.

For meaningful comparison, we have only included results based on fully connected feedforward networks and exclude convolutional networks, as currently there is no available code infrastructure for convolution kernel for covariance computation. While generalizing the standard convolution kernel to second-order is conceptually straightforward, its efficient numerical implementation is better suited for a future study. For this reason, we have not included comparisons with CIFAR-10 as this dataset is most commonly solved using convolution networks (1–3). We also restrict our comparison to models using rate coding and exclude those using time coding or rank-order coding (2, 4). We have included comparisons to gradient-based learning methods, both conversion-based and direct, and excluded comparisons to methods using spike-time dependent plasticity.

While we can't directly compare the training results to that presented in ref. (5), as they consider exclusively convolutional neural networks, it is instructive to compare the general approach and the theories (which is independent of the specific model architecture). In brief, the authors in ref.(5) analyzed the relationship between the sample spike count and the input rate, and identified the source of error during ANN-to-SNN conversion (see Eq. 5 of their paper). They then showed how various techniques (specifically, weight normalization, using subtraction rather than hard reset in SNN, and using analog input instead of Poisson spikes) can be used to improve ANN-to-SNN conversion by eliminating this conversion error. The essential difference of this approach from ours is that these techniques are applied after training is done, whereas our model does not require any post-training optimization. This is because the MNN represents a *tight* approximation to SNN in the first place. In other words, the conversion error is already minimized before training, if not completely eliminated. This is why we refer our approach as 'reconstruction' of SNN from MNN as opposed to 'conversion' of ANN to SNN. However, it is important to point out that our method does not exclude post-training optimization. In the future, both techniques can be applied in tandem to further boost performance in SNN.

There are a number of benefits by considering probabilistic coding, both in terms of learning and inference. For learning, by considering the second-order moments of the spiking neurons, the immediate benefit is that we obtain the MNN as a tight approximation to the SNN, allowing us to reconstruct the SNN without any post-training optimization (as pointed out above). Another benefit is that by considering the stationary statistics, the MNN avoids the need to deal with temporal dynamics and thus does not require backpropagation-through-time. For inference, our approach can reduce the variance in the model prediction by manipulating correlated neural activity (in line with theories of neural population code), and in turn improve inference speed.

2. Model settings for larger datasets

The Fashion-MNIST dataset consists of grayscale images of clothing items belonging to 10 categories, with 60,000 images for training and 10,000 images for validation. The CIFAR-10 dataset consists of natural images belonging to 10 categories, with 50,000 training images and 10,000 validation images. We adopt a feedforward model configuration similar to the one used for the MNIST dataset, and encode the pixel intensities of each image into Poisson spike trains. For the Fashion-MNIST dataset, our models consist of an input layer with 784 neurons, a hidden layer with 1000 neurons, and a linear readout with 10 units. For the CIFAR-10 dataset, the models consist of an input layer with 3072 neurons, three hidden layers (each with 1000 neurons), and a linear readout with 10 units. We train MNN models on these datasets with three different loss functions: the moment cross-entropy (MCE) loss with readout times $\Delta t = 1$ or 10 ms, and the standard cross-entropy (CE) loss. As a reference for comparison, we also train conventional artificial neural networks (ANNs) with ReLU activation and identical network architectures (using CE loss). In both tasks, we employ AdamW with default hyperparameters (a learning rate of 0.001 and a weight decay of 0.01) to train these models for 30 epochs and repeat the experiments 5 times. Furthermore, we reconstruct spiking neural networks (SNNs) from the trained MNN models and evaluate the classification accuracy of the corresponding SNNs by running simulations for 100 ms with a time increment of $\delta t = 1$ ms, averaged over 100 trials.

3. Comparison of spike count distributions between MNN and SNN

To further demonstrate that the MNN faithfully captures the distributions of spiking activity of the reconstructed SNNs, we compare the spike count distribution obtained from simulations of the SNN to that predicted by the MNN. To do so, we run simulations of the SNN to perform inference on the MNIST dataset, using the same model structure as presented in the main text, and record the spike count of hidden layer neurons over a 1000 ms time window across 500 trials. For this purpose, a time step of $\delta t = 0.01$ ms is used for simulating the SNN.

Figure S2**a** shows the spike count distributions of 10 representative neurons (out of 1000 hidden neurons) for a randomly picked input image. The neurons shown cover a wide range of mean firing rate and Fano factors. Specifically, we first group the neurons according to their mean firing rates (five bins in the range of 0-150 Hz) and Fano factors (five bins in the range of 0-1), and then pick a random neuron from each group. The panels in Figure S2**a** are organized in a way that the mean firing rates increase from top to bottom whereas the Fano factors increase from left to right, with each row corresponding to neurons with similar mean firing rate and each column to neurons with similar Fano factors. Note that no neuron is found with both a high firing rate and a high Fano factor. We find that the spike count histograms as obtained from simulations of the SNN match well to the predictions by the MNN (orange curves, which show gamma distributions with mean and variance given by the MNN).

To show the MNN also captures the joint distributions, we show the joint histograms between pairs of neurons and compare them to the prediction by the MNN. Figure S2**b** and Fig. S2**c** show the marginal joint probability density functions of the spike counts of pairs of neurons with similar mean firing rates (neuron 0, 1, 2, 3, 4) and similar Fano factors (neuron 0, 5, 8, 9, 10), respectively. The heat maps indicate the histograms obtained from simulations of the SNN and the orange ellipses indicate the covariance calculated by the MNN (scaled to 2 units of standard deviation).

4. Analysis of backpropagation in the moment neural network

In gradient-based learning, what differentiates one learning algorithm from another is how the gradient $\partial L/\partial\theta$ is estimated. It is therefore instructive to analyze how backpropagation is computed in MNN and how it differs from other methods. Consider a feedforward network with n hidden layers, with each hidden layer consisting the components shown in Fig. 2**b**. For compactness, denote the output of the k-th hidden layer as $\Lambda^{(k)} = (\mu^{(k)}, C^{(k)})$ which groups together the mean $\mu^{(k)}$ and covariance $C^{(k)}$ of neural activity into a single vector with $\frac{1}{2}N(N+1)$ independent elements. The gradient of the loss function with respect to the model parameters $\theta^{(k)}$, which encompasses the synaptic weights $w^{(k)}$ and the scale and bias parameters $\gamma^{(k)}$, $\beta^{(k)}$, and $C^{\text{ext},(k)}$, can be expressed as

$$\frac{\partial L}{\partial \theta^{(k)}} = \frac{\partial L}{\partial \Lambda^{(n)}} \left[\frac{\partial \Lambda^{(n)}}{\partial \Lambda^{(n-1)}} \frac{\partial \Lambda^{(n-1)}}{\partial \Lambda^{(n-2)}} \cdots \frac{\partial \Lambda^{(k+1)}}{\partial \Lambda^{(k)}} \right] \frac{\partial \Lambda^{(k)}}{\partial \theta^{(k)}}. \quad [1]$$

The gradient of each hidden layer can be further written out as

$$\frac{\partial \Lambda^{(n)}}{\partial \Lambda^{(n-1)}} = \begin{bmatrix} \frac{\partial \mu^{(n)}}{\partial \bar{\mu}^{(n)}} & \frac{\partial \mu^{(n)}}{\partial \bar{C}^{(n)}} \\ \frac{\partial C^{(n)}}{\partial \bar{\mu}^{(n)}} & \frac{\partial C^{(n)}}{\partial \bar{C}^{(n)}} \end{bmatrix} \begin{bmatrix} \frac{\partial \bar{\mu}^{(n)}}{\partial \hat{\mu}^{(n)}} & \frac{\partial \bar{\mu}^{(n)}}{\partial \hat{C}^{(n)}} \\ \frac{\partial \bar{C}^{(n)}}{\partial \hat{\mu}^{(n)}} & \frac{\partial \bar{C}^{(n)}}{\partial \hat{C}^{(n)}} \end{bmatrix} \begin{bmatrix} \frac{\partial \hat{\mu}^{(n)}}{\partial \mu^{(n-1)}} & 0 \\ 0 & \frac{\partial \hat{C}^{(n)}}{\partial C^{(n-1)}} \end{bmatrix}, \quad [2]$$

where each block, from left to right, represents the Jacobian matrix of the moment activation, the moment batchnormalization, and the synaptic summation, respectively. In practice, the backpropagation is implemented using Pytorch's efficient autograd functionality, with custom gradients for the moment activation.

Specifically, the gradients for the moment activation [equations (13)-(15) in the main text] are

$$\begin{cases} \frac{\partial L}{\partial \bar{\mu}_i} = \frac{\partial L}{\partial \mu_i}\frac{\partial \mu_i}{\partial \bar{\mu}_i} + \frac{\partial L}{\partial \sigma_i}\frac{\partial \sigma_i}{\partial \bar{\mu}_i} + 2\frac{\partial \chi_i}{\partial \bar{\mu}_i}\Gamma_i & [3] \\ \frac{\partial L}{\partial \bar{\sigma}_i} = \frac{\partial L}{\partial \mu_i}\frac{\partial \mu_i}{\partial \bar{\sigma}_i} + \frac{\partial L}{\partial \sigma_i}\frac{\partial \sigma_i}{\partial \bar{\sigma}_i} + 2\frac{\partial \chi_i}{\partial \bar{\sigma}_i}\Gamma_i, & [4] \\ \frac{\partial L}{\partial \bar{\rho}_{ij}} = \frac{\partial L}{\partial \rho_{ij}}\chi_i\chi_j, \quad i \neq j & [5] \end{cases}$$

where

$$\Gamma_i = \sum_{l \neq i} \left(\frac{\partial L}{\partial \rho_{il}} \rho_{il} \right) \chi_l. \quad [6]$$

Here, $\frac{\partial L}{\partial \mu_i}$, $\frac{\partial L}{\partial \sigma_i}$ and $\frac{\partial L}{\partial \rho_{ij}}$ are the inputs of the backward pass, $\frac{\partial L}{\partial \bar{\mu}_i}$, $\frac{\partial L}{\partial \bar{\sigma}_i}$ and $\frac{\partial L}{\partial \bar{\rho}_{ij}}$ are the outputs of the backward pass. The derivatives of μ_i, σ_i and χ_i with respect to $\bar{\mu}_i$, $\bar{\sigma}_i$ are evaluated using the formulae provided in (6).

The general form of backpropagation for the MNN, as revealed by equation (1) and (2), is directly analogous to the backpropagation for rate-based ANNs, as MNN is a natural generalization of ANN to second-order moments. The key difference is that the gradients not only flow through the mean firing rate μ but also the covariance C. To illustrate the idea, consider the Jacobian matrix for the moment activation. There are two ways covariance contributes to the gradient estimation, one of which is through terms directly containing partial derivatives of, or with respect to, covariance (i.e. $\frac{\partial \mu}{\partial \bar{C}}$, $\frac{\partial C}{\partial \bar{\mu}}$, and $\frac{\partial C}{\partial \bar{C}}$), and the other through indirect augmentation of the partial derivatives involving only the mean $\frac{\partial \mu}{\partial \bar{\mu}}$. In the latter, the role of the input covariance can be interpreted as dynamically controlling the steepness of the mean activation (as can be seen from Fig. 2**b**), with a larger input variance leading to a smoother gradient.

It is also interesting to compare equation (1)-(2) with direct gradient estimation based on the temporal dynamics of SNN. The most widely adopted approach is backpropagation through time (BPTT) applied directly to the membrane potential dynamics (7–11). These methods typically involve surrogate gradients for dealing with discontinuous jumps of membrane potential. However, BPTT and its variants for training SNN are best suited for discrete-time SNN models. For continuous-time SNN models, the neuronal dynamics must first be discretized and unwrapped in time, which may lead to higher cost when finer

temporal resolution is required. Another approach to evaluating the gradient is through spike time coding (4, 12), allowing for exact gradient to be computed. The moment embedding approach presented here provides a way to train continuous-time SNN, without explicitly dealing with temporal dynamics.

5. Comparison to existing approaches to training spiking neural networks

The moment embedding developed in this study leads to a novel approach to training spiking neural networks (SNN) under the principle of stochastic neural computing (SNC). Here, we briefly review existing approaches to training SNNs and compare them to ours.

Existing approaches to training SNN can largely fall into two categories: ANN-to-SNN conversion and direct SNN training methods (13, 14). In ANN-to-SNN conversion, a standard artificial neural network (ANN) is pre-trained using traditional backpropagation algorithms, and then converted to an SNN model by mapping the ANN parameters onto SNN. These rate-coded network conversion often results in accuracy loss and relatively high inference latency. Many works have focused on transferring weights from ANN with continuous-valued activation to SNN and setting the firing threshold of neurons layer by layer to achieve near-lossless performance but poor latency (5, 15, 16); other approaches have also been proposed to improve the accuracy while trying to reduce the inference time steps and keep the event-driven sparsity for better energy efficiency (17).

Direct approaches to training SNN, on the other hand, use backpropagation-through-time to optimize the parameters of an SNN directly (14, 18, 19). This requires formulating the SNN as an equivalent recurrent neural network with binary spike inputs. This enables emergent spike-timing codes which convey information with the relative timing between spikes for efficient sparse coding. The main challenge of direct training is due to the discontinuous, non-differentiable nature of spike generation. Diverse methods have been proposed to solve the problem by defining surrogate gradient or spike time coding to enable backpropagation; some examples include spike layer error reassignment (SLAYER) (8), spatiotemporal backpropagation (STBP) (9), and local online learning via eligibility propagation (e-prop) (11). In general, direct training approaches tend to achieve fewer spikes, lower latency and energy consumption, but need to overcome difficulties on convergence and performance degradation as network size increases (14).

Our approach represents significant advances over previous methods, both conceptual and practical. Conceptually, the moment embedding approach overcomes previous challenges of mapping continuous-valued neural network models to their spiking counterparts by deriving the moment representation of spiking neural activity from first principles. As a consequence, the MNN can serve as an effective proxy for training SNN, not by *conversion* but by *reconstruction*, as the MNN represents a *tight* approximation to SNN under the view of probabilistic coding. The practical benefit this brings about is that no extra parameters are introduced both before and after training. As illustrated in Fig. 2**b**, the only trainable parameters in the MNN are the synaptic weights, and the scaling factors and biases in the moment batch normalization. Notably, the dimensionality of these trainable parameters are identical to its ANN-counterpart. We find that the performance of MNN is comparable to its ANN counterpart under the same network structures (see table 2 in the main text). In contrast, ANN-to-SNN conversion requires extra parameters or various post-training optimization techniques to eliminate conversion error, such as manually adjusting the firing threshold and the scaling of firing rate at the input layer (15), modifying or adding trainable parameters to the activation function (1, 3, 16), weight scaling (20) and weight normalization (5, 15). We point out that our method does not prohibit post-training optimizations and both techniques can be applied in tandem in the future to further boost the performance of the SNN. Additionally, ANN-to-SNN conversion is often criticized for its reliance on rate coding that leads to slow convergence in the converted SNN. It has been suggested that post-training weight normalization could improve the convergence (15). Here, by generalizing ANN to second-order moments, the MNN enables the representation of uncertainty and subsequently reduction of the inference time through manipulating correlated neural variability. Our approach thus provides not only an alternative way to train SNN but also insights to how it operates through the lens of stochastic neural computing. See table S1 for a quantitative comparison of performance between different methods.

A number of studies have explored how noise can be exploited for various benefits during learning by using simplified probabilistic spiking neural network models (21–23). By modeling spike generation as a Bernoulli process with firing probability depending on the neuron's input, it has been shown that a three-factor learning rule can be recovered from a probability learning objective (22). Using simplified binary neural network with stochastic synapses, it has been shown that multiplicative synaptic noise can lead to intrinsic weight normalization during learning (24). However, these simplified models are limited to uncorrelated neural spiking activity. By exploiting the spiking neuron's innate ability to nonlinearly couple signal and noise, our work demonstrates for the first time the learning and implementation of stochastic neural computing in spiking neural networks with correlated neural variability, and highlights how noise correlation can be exploited to reduce prediction uncertainty and in turn enhance inference speed.

6. Role of hidden neurons as accuracy and precision contributors

In the main text, we have demonstrated a moment closure approach to training SNN. To better understand the functional role of correlated variability and underlying computational processes, we now turn to analyzing the coding properties of the trained network.

To understand how the activity states of hidden layer neurons affect task performance, we can think of how they shape the geometry of the readout distribution relative to the decision boundary. If an infinite readout time is allowed, the situation is simple as the only goal is to make the readout mean lie within the domain of correct prediction. However, for any finite readout

time, the readout is a distribution so we want to maximize the probability mass for correct predictions. Under this scenario, we can either shift the readout mean or shape the readout covariance so that the readout distribution overlaps with the domain of correct prediction as much as possible. Figure S1**a** shows a conceptual illustration of different ways the readout distribution can be manipulated to influence inference accuracy and precision. Here, we focus our analysis on the those input images that are correctly classified. Note that only the components perpendicular to the decision boundary affect the probability mass of correct predictions.

Since the readout is linear we can straightforwardly inspect the contributions from individual neurons or neuronal pairs to the readout moments. Specifically, the contribution from neuron k to the readout mean is $w_{ik}\mu_k$ and the contribution from neuronal pair (k,l) to the readout covariance is $w_{ik}C_{kl}w_{jl}$, where w_{ik} are the readout weights and μ_l and C_{kl} are mean and covariance of hidden neurons. In all proceeding analysis, the readout index i is ranked such that the readout mean $\hat{\mu}_i = \sum_k w_{ik}\mu_k$ is in descending order. To further simplify, we focus on the readout dimensions with the largest and the second largest means and consider the marginal distribution within this two dimensional space. This is justified as long as a specific readout distribution does not overlap significantly with the other class domains. As we have shown in Fig. S1**a**, only contributions in the direction perpendicular to the decision boundary affects task performance. Therefore, we can project individual contributions to the readout along the direction $v = (1,-1)/\sqrt{2}$. This leads to the scalar quantities

$$\delta\hat{\mu}_k^{\perp} = \frac{1}{\sqrt{2}}(w_{1k} - w_{2k})\mu_k, \qquad [7]$$

and

$$\delta\hat{\sigma}_{kl}^{2,\perp} = \frac{1}{2}[w_{1k}C_{kl}w_{1l} + w_{2k}C_{kl}w_{2l} - 2w_{1k}C_{kl}w_{2l}], \qquad [8]$$

which we refer to as *normal contributions* to readout mean and variance, respectively. Assuming that $i = 1$ corresponds to the correct prediction, then a positive or negative $\delta\hat{\mu}_i^{\perp}$ would imply a positive or negative contribution to inference accuracy, respectively. Similarly, a negative $\delta\hat{\sigma}_{kl}^{2,\perp}$ would imply a reduction in uncertainty and thus an improvement to inference precision. For the readout variance, tracking contributions from all neuronal pairs is a daunting task so we calculate instead the overall contribution by an individual neuron as

$$\delta\hat{\sigma}_k^{2,\perp} = \delta\hat{\sigma}_{kk}^{2,\perp} + \frac{1}{2}\sum_{l\neq k}\delta\hat{\sigma}_{kl}^{2,\perp}. \qquad [9]$$

Similar analysis can be performed for incorrectly classified images by considering the marginal readout distribution on the dimensions corresponding to the target class and the highest mean read out, with the role of covariane reversed.

Using these measures, we can now systematically categorize hidden layer neurons based on their contributions to task performance. Again, we restrict our analysis below to correctly classified input images. To provide an overview, for each hidden neuron, we calculate the normal contributions averaged over all input images, denoted as $\langle\delta\hat{\mu}_k^{\perp}\rangle$ and $\langle\delta\hat{\sigma}_k^{2,\perp}\rangle$, respectively. Next, we map all hidden neurons to the plane spanned by $\langle\delta\hat{\mu}_k^{\perp}\rangle$ and $\langle\delta\hat{\sigma}_k^{2,\perp}\rangle$, as shown by the scatter plots in Fig. S1**b**. The average contributions over all neurons are found to be 0.0057 for readout mean and 0.0031 for readout variance, as indicated by the lines in Fig. S1**b**. We can then broadly categorize a hidden neuron as an *accuracy contributor* if its normal contribution to readout mean is above average and a *precision contributor* if its normal contribution to readout variance is below average. We find that among 1000 hidden neurons, 480 are accuracy contributors, 567 are precision contributors, 214 are simultaneously an accuracy contributor and a precision contributor, and 167 are neither.

Given this overview, we next analyze a neuron's stimulus selectivity by calculating the average contributions to readout mean and variance over images belonging to a specific class c, denoted as $\langle\delta\hat{\mu}_k^{\perp}\rangle_c$ and $\langle\delta\hat{\sigma}_k^{2,\perp}\rangle_c$, respectively. To provide some intuition, we first present the normal contributions to readout mean and variance by a representative neuron (neuronal index 5), as shown in Fig. S1**c**. This neuron's selectivity for certain image classes can be clearly seen. The average contributions for each image class are summarized in Fig. S1**d**. The lines mark the global average calculated from all images and all neurons as in Fig. S1**b**. It can be seen that this neuron's contributions to readout mean are above global average for image class '0' and '1', whereas its contributions to readout variance are below global average for all input class except '7' and it even reduces uncertainty for image class '9'. This example demonstrate that the role of a neuron as an accuracy or a precision contributor may change depending on the input class. By repeating this analysis for all neurons, we construct heatmaps showing their contributions in classifying different input classes, as shown in Fig. S1**e**. Here, 100 representative neurons are shown, evenly spaced according to their rank calculated from their overall contributions to readout mean. In general, the neural code in the trained network is highly distributed such that each neuron can play multiple roles across different input classes.

7. Validity of moment closure up to second order

A question arises regarding the validity of moment closure with only the first and second order moments. As we have shown, the spike count distributions obtained from simulations of the SNN well match to that predicted by the MNN (see Fig. S2), indicating the validity of using second-order moments for modeling stochastic neural dynamics. The validity of this approach is also supported by experimental and theoretical evidence. It has been found that the collective behavior exhibited by the neural population in the vertebrate retina is well accounted for by a model that captures the observed pairwise correlations but assumes no higher-order interactions (25). A theoretical study investigates the effect of higher-order moments in a stochastic binary neural network model (26) and has found that in a weakly correlated state, the contributions from higher-order moments

to the network dynamics are significantly smaller than pairwise interactions. This suggests that the first two moments are quite sufficient for modeling the stochastic dynamics of neural activity. While second-order moments do not provide the complete information about a joint probability distribution, they nonetheless provides a rich description of of fluctuating neural activity as compared to uncorrelated Poisson or binary neurons commonly assumed in the literature.

8. Spiking neural network implementation on Loihi

Loihi is a digital, event-driven asynchronous circuit chip implementing discrete-time spiking neural network model (27–29). Each Loihi chip contains 128 neuromorphic cores and 3 low-power embedded x86 CPU cores. Every neuromorphic core has up to 1K leaky integrate-and-fire (LIF) neurons, implementing a total of 128K neurons and 128M synaptic connections per chip for spike-based neural computing. Embedded CPU cores are mainly used for management as well as bridging between conventional and neuromorphic hardware. Single Loihi chip could show orders of magnitude improvements in terms of low power consumption and fast processing speed on certain tasks compared with conventional hardware. Loihi implements a discretized version of the LIF neuron model as follows

$$V_i(t) = (1 - \tfrac{1}{\tau_v})V_i(t-1) + I_i(t) + \beta, \tag{10}$$

$$I_i(t) = (1 - \tfrac{1}{\tau_u})I_i(t-1) + \sum_j w_{ij} s_j(t), \tag{11}$$

where $V_i(t)$ and $I_i(t)$ represent the membrane potential and synaptic current received by the ith neuron at a discrete time step t, respectively, and $s_j(t)$ represents binary spike trains of pre-synaptic neurons. The quantities τ_v, τ_u, and β represent the membrane time constant, synaptic time constant, and bias current, respectively.

The following parameter setting is used to match the discrete LIF model [equation (11)] implemented by Loihi to the continuous LIF model [equation (6) in the main text]. First, the synaptic current time constant τ_u is set to be infinite so that the synaptic current becomes a weighted sum of input spikes. Second, we use exact solution of the subthreshold membrane potential rather than the Euler scheme for numerical integration to accommodate larger simulation time step δt, which is often desirable for saving energy on hardware. As a result, the membrane time constant is set to be $\frac{1}{\tau_v} = 1 - e^{-L\delta t}$ and the bias current is set to be $\beta_i = \frac{1}{L}(1 - e^{-L\delta t})I_i^{\mathrm{ext}}$, where I_i^{ext} is the external input current in the continuous model. By default, we set $L = 0.05\ \mathrm{ms}^{-1}$ and $\delta t = 1$ ms.

Since Loihi only supports 9-bit signed integer representation of synaptic weights taking values in the range $[-256, 254]$, appropriate rescaling is needed to map the synaptic weights from physical units to this 9-bit representation. Specifically, a factor of $k = \frac{254}{\max(|\bar{w}^{\mathrm{MNN}}|)}$ is used to scale the synaptic weight, the membrane potential and the bias currents, which are rounded down to integer values afterward.

One nuance regarding the hardware implementation is how to emulate the linear readout [equation (**??**)] used in the continuous model. This is achieved with an output layer consisting of perfect integrator neurons ($L = 0$) with infinite firing threshold. Under this setting, the linear readout y is equivalent to the membrane potential in the output layer divided by the readout time T, which can be recorded by the hardware. The model prediction is based on the output neuron with the highest membrane potential.

Performance of hardware implementation of an SNN is typically characterized by a number of key metrics including accuracy, energy consumption, and latency (also known as delay). Accuracy measures the fraction of correctly predicted samples in the dataset averaged across trials. Energy consumption measures the dynamic energy consumed by the neuromorphic cores per inference, whereas delay measures the elapsed wall time per inference. Both energy consumption and delay are measured by the on-board probe of Loihi Nahuku-32 board using NxSDK version 2.0.

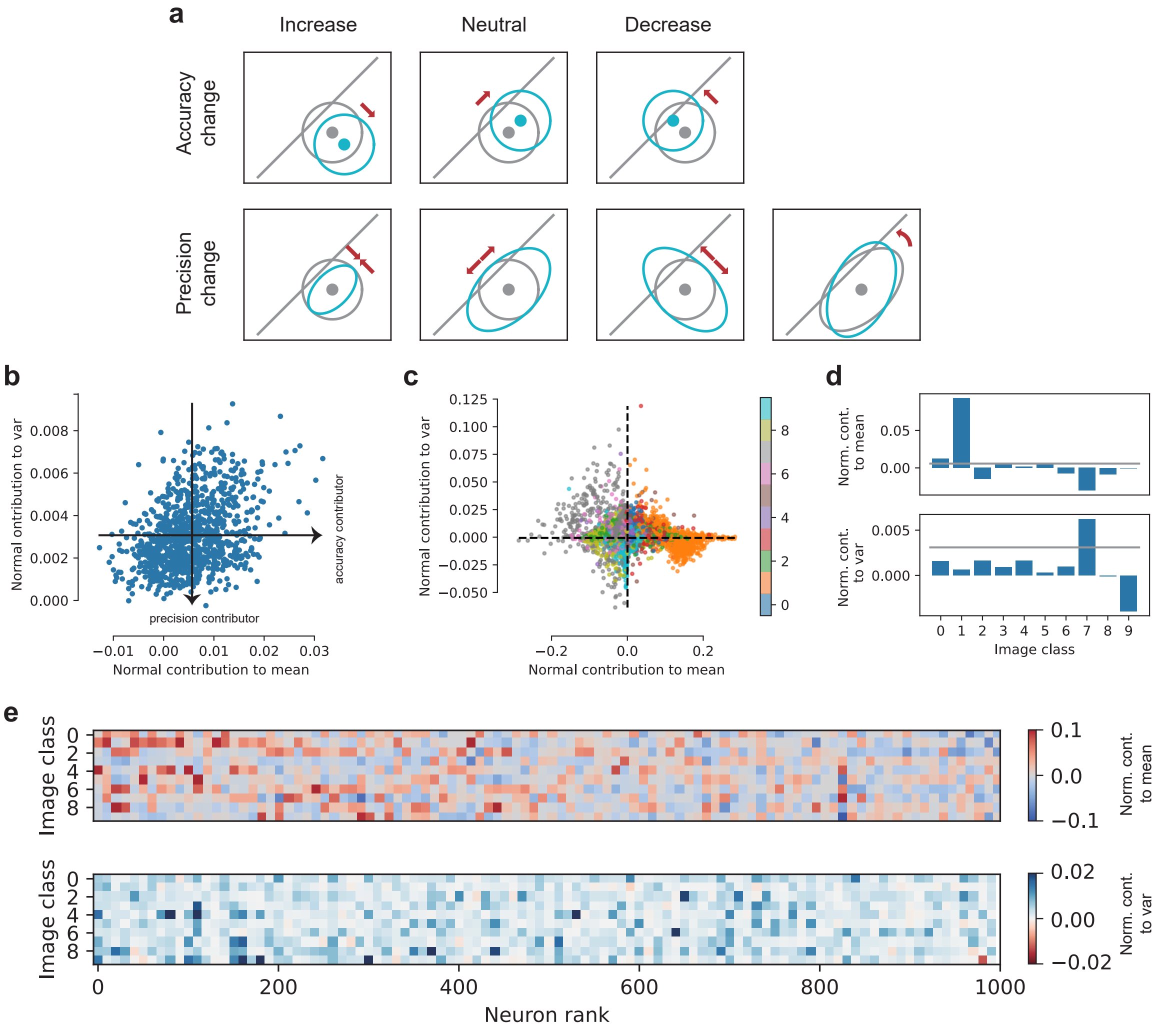

Fig. S1. Coding properties of hidden neurons. **a**, Illustration of different ways readout mean and covariance influence task performance. Solid line indicates the decision boundary, the area below which corresponds to correct predictions. Dots and ellipses indicate mean and covariance of readout distributions, respectively. **b**, Normal contributions to readout mean and variance averaged over all input images. Each dot corresponds to a hidden neuron. **c**, Normal contributions to readout mean and variance by one hidden neuron. Each dot corresponds to an input image and colors represent image classes. **d**, Normal contributions in **c** averaged over all input images in each class. **e**, Normal contributions averaged over all input images for 100 representative neurons evenly spaced according to their rank calculated from the normal contributions to readout mean shown in **b**.

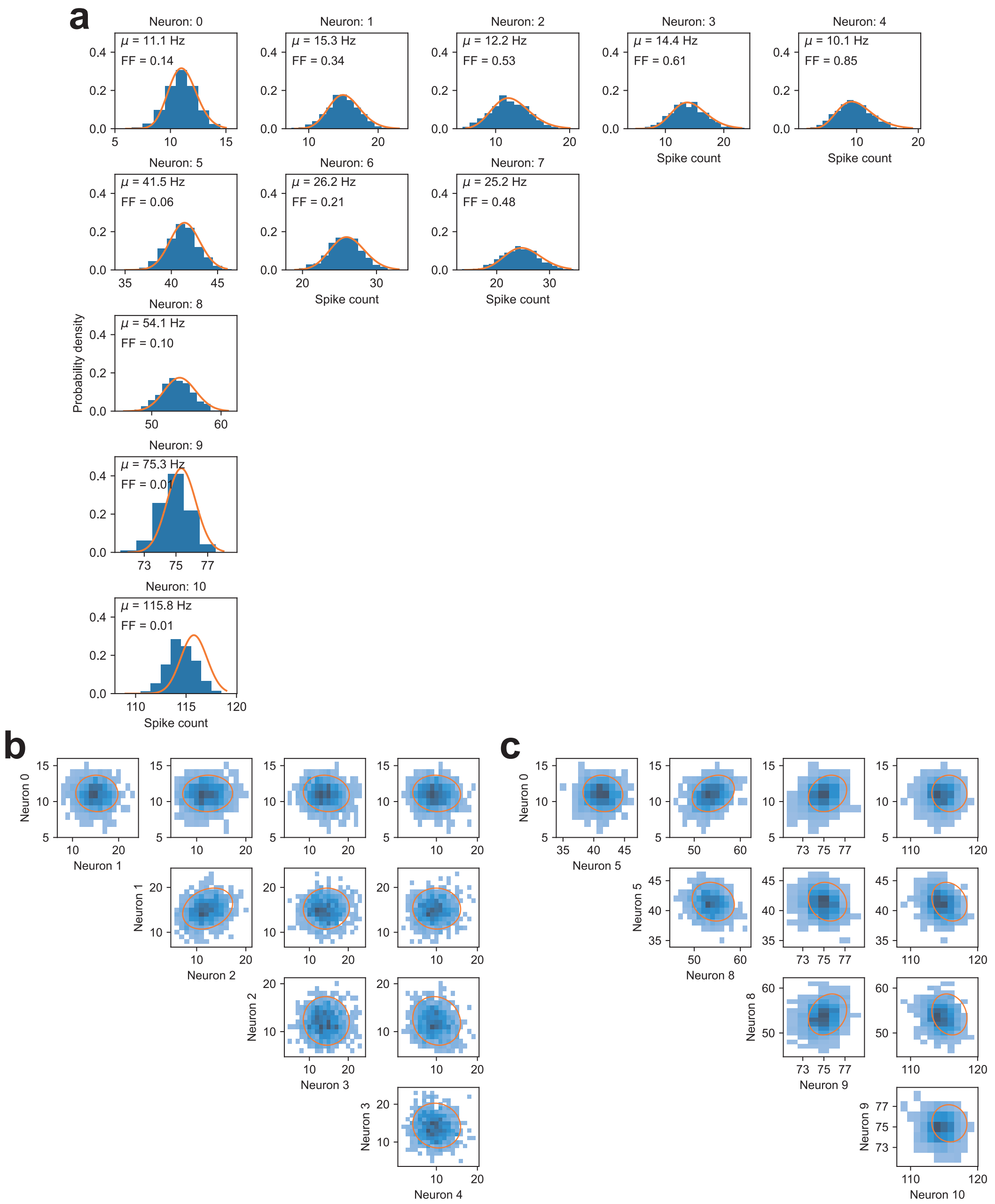

Fig. S2. Comparing spike count distributions between MNN and SNN. **a**, Spike count distribution of representative neurons in the hidden layer. Histograms corresponds to spike counts obtained from simulations of the SNN over 1000 ms time window across 500 trials. Orange curve shows gamma distributions with mean and variance calculated from the MNN. **b**, Marginal joint probability density of the spike counts of pairs of neurons with similar mean firing rates (neuron 0, 1, 2, 3, 4). **c**, Marginal joint probability density of the spike counts of pairs of neurons with similar Fano factors (neuron 0, 5, 8, 9, 10). Heat map indicates the probability density obtained from simulations of the SNN; orange ellipse indicates covariance calculated by the MNN (scaled to 2 standard deviations).

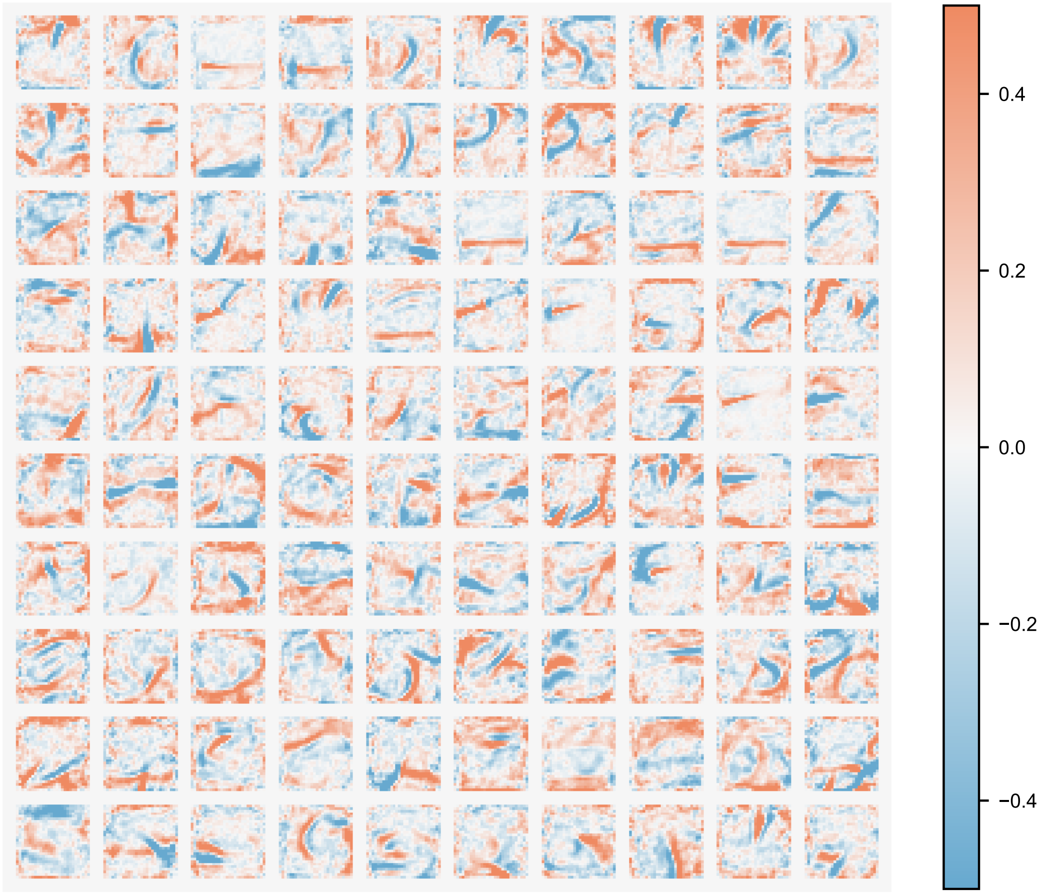

Fig. S3. Illustration of feedforward weights received by individual hidden neurons. Weights to 100 representative neurons are shown, ranked according to their normal contributions to readout mean averaged over all input images. The ranking proceeds from left to right, and top to bottom, from the highest normal contribution to the lowest.

Table S1. Comparison of different methods for training spiking neural network on the MNIST dataset. IF indicates integrate-and-fire model without leak.

	Network structure	Accuracy % (ANN/SNN)	Inference time (ms)	#spikes	τ_{m} (ms)	V_{th} (mV)	Techniques
**Conversion**							
Hunsberger et al. (3)	784-500-200-10	-/98.37	n/a	n/a	20	20	Activation smoothing; Additive noise
Diehl et al. (15)	784-1200-1200-10	98.68/ 98.64	6†	1146*	IF	1	Weight normalization
		98.68/ 98.48	200*	2525*	IF	4	Manual adjustment of firing threshold
**Direct**							
Lee et al. (19)	784-800-10	-/98.71	1000	n/a	20	adaptive	BPTT with error normalization; weight and threshold regularization
Wu et al. (9)	784-800-10	-/98.89	30	n/a	0.1 ms	1.5	STBP
Yan et al. (2)	784-256-64-10	-/98.5	10	1148	1.11 ms	1.5	G-STBP; Sparsity regularization
**Reconstruction**							
MNN (this study)	784-1000-10	98.45/ 98.43	6.6†	985 (300 in hidden layer)	20 ms	20	Moment embedding

* Estimated from Fig. 2 of their paper.

† At 99.6% of maximum accuracy.

References

1. J Ding, Z Yu, Y Tian, T Huang, Optimal ANN-SNN conversion for fast and accurate inference in deep spiking neural networks (2021).
2. Y Yan, et al., Graph-based spatio-temporal backpropagation for training spiking neural networks in *2021 IEEE 3rd International Conference on Artificial Intelligence Circuits and Systems (AICAS)*. pp. 1–4 (2021).
3. E Hunsberger, C Eliasmith, Spiking deep networks with LIF neurons (2015).
4. TC Wunderlich, C Pehle, Event-based backpropagation can compute exact gradients for spiking neural networks. *Sci. Reports* **11**, 12829 (2021).
5. B Rueckauer, IA Lungu, Y Hu, M Pfeiffer, SC Liu, Conversion of continuous-valued deep networks to efficient event-driven networks for image classification. *Front. Neurosci.* **11** (2017).
6. Y Qi, Moment neural network and an efficient numerical method for modeling irregular spiking activity. *Phys. Rev. E* **110**, 024310 (2024).
7. EO Neftci, H Mostafa, F Zenke, Surrogate gradient learning in spiking neural networks: Bringing the power of gradient-based optimization to spiking neural networks. *IEEE Signal Process. Mag.* **36**, 51–63 (2019).
8. SB Shrestha, G Orchard, SLAYER: Spike layer error reassignment in time. *Adv. Neur. In.* **31** (2018).
9. Y Wu, L Deng, G Li, J Zhu, L Shi, Spatio-temporal backpropagation for training high-performance spiking neural networks. *Front. Neurosci.* **12** (2018).
10. C Lee, SS Sarwar, P Panda, G Srinivasan, K Roy, Enabling spike-based backpropagation for training deep neural network architectures. *Front. Neurosci.* **14** (2020).
11. G Bellec, et al., A solution to the learning dilemma for recurrent networks of spiking neurons. *Nat. Commun.* **11**, 3625 (2020).
12. SM Bohte, JN Kok, H La Poutré, Error-backpropagation in temporally encoded networks of spiking neurons. *Neurocomputing* **48**, 17–37 (2002).
13. K Roy, A Jaiswal, P Panda, Towards spike-based machine intelligence with neuromorphic computing. *Nature* **575**, 607–617 (2019).
14. M Pfeiffer, T Pfeil, Deep learning with spiking neurons: opportunities and challenges. *Front. Neurosci.* **12**, 774 (2018).
15. PU Diehl, et al., Fast-classifying, high-accuracy spiking deep networks through weight and threshold balancing in *2015 International Joint Conference on Neural Networks (IJCNN)*. pp. 1–8 (2015).
16. Z Yan, J Zhou, WF Wong, Near lossless transfer learning for spiking neural networks. *Proc. AAAI Conf. on Artif. Intell.* **35**, 10577–10584 (2021).
17. N Rathi, G Srinivasan, P Panda, K Roy, Enabling deep spiking neural networks with hybrid conversion and spike timing

dependent backpropagation (2020).
18. Y Yan, et al., Backpropagation with sparsity regularization for spiking neural network learning. *Front. Neurosci.* **16**, 760298 (2022).
19. JH Lee, T Delbruck, M Pfeiffer, Training deep spiking neural networks using backpropagation. *Front. Neurosci.* **10**, 508 (2016).
20. R Kim, Y Li, TJ Sejnowski, Simple framework for constructing functional spiking recurrent neural networks. *Proc. Natl. Acad. Sci.* **116**, 22811–22820 (2019).
21. G Ma, R Yan, H Tang, Exploiting noise as a resource for computation and learning in spiking neural networks. *Patterns* p. 100831 (2023).
22. H Jang, O Simeone, B Gardner, A Grning, An introduction to probabilistic spiking neural networks. *IEEE Signal Proc. Mag.* **36**, 64–77 (2019).
23. S Dutta, et al., Neural sampling machine with stochastic synapse allows brain-like learning and inference. *Nat. Commun.* **13**, 2571 (2022).
24. G Detorakis, et al., Inherent weight normalization in stochastic neural networks in *Advances in Neural Information Processing Systems*, eds. H Wallach, et al. Vol. 32, (2019).
25. E Schneidman, MJ Berry, R Segev, W Bialek, Weak pairwise correlations imply strongly correlated network states in a neural population. *Nature* **440**, 1007–1012 (2006).
26. D Dahmen, S Grün, M Diesmann, M Helias, Second type of criticality in the brain uncovers rich multiple-neuron dynamics. *Proc. Natl. Acad. Sci.* **116**, 13051–13060 (2019).
27. M Davies, et al., Advancing neuromorphic computing with Loihi: a survey of results and outlook. *Proc. IEEE* **109**, 911–934 (2021).
28. M Davies, et al., Loihi: a neuromorphic manycore processor with on-chip learning. *IEEE Micro* **38**, 82–99 (2018).
29. A Renner, F Sheldon, A Zlotnik, L Tao, A Sornborger, The backpropagation algorithm implemented on spiking neuromorphic hardware (2021).
